# Thermoplastic Starch Composites with Highly Exfoliated Nano-Clay Fillers and Excellent Barrier Properties

**DOI:** 10.3390/ma19020347

**Published:** 2026-01-15

**Authors:** Veronika Gajdosova, Beata Strachota, Vaclav Pokorny, Libuse Brozova, Jan Kozisek, Ewa Pavlova, Zdenek Stary, Miroslav Slouf, Adam Strachota

**Affiliations:** 1 Institute of Macromolecular Chemistry, Czech Academy of Sciences, Heyrovsky Sq. 2, 162 06 Prague 6, Czech Republic; gajdosova@imc.cas.cz (V.G.); beata@imc.cas.cz (B.S.); pokorny@imc.cas.cz (V.P.); brozova@imc.cas.cz (L.B.); kozisek@imc.cas.cz (J.K.); pavlova@imc.cas.cz (E.P.); stary@imc.cas.cz (Z.S.); 2 Department of Physical and Macromolecular Chemistry, Faculty of Science, Charles University, Hlavova 2030, 128 40 Prague 2, Czech Republic

**Keywords:** thermoplastic starch, biodegradable nanocomposite, clay, laponite, montmorillonite

## Abstract

Thermoplastic starch (TPS) nanocomposites with unprecedentedly high loadings of up to 15 wt.% of the nano-clays Laponite (LAP; a synthetic product capable of good dispersion in suitable media) or Montmorillonite (MMT; modified with dialkyldimethylammonium chloride) were prepared by means of our new, two-step TPS preparation protocol. In both the TPS/LAP and TPS/MMT composites, we achieved perfect dispersion and extensive exfoliation of the nano-clays, resulting in pronounced improvements in mechanical performance (modulus increased up to one order of magnitude) and in excellent gas-barrier properties (extremely small permeabilities for O_2_, CO_2_, and even H_2_). MMT, owing to its larger platelet size and to the formation of partially exfoliated multi-layer structures, generated a percolating filler network that provided particularly strong reinforcement, especially at 15 wt.% loading. LAP, though more completely exfoliated, generated a somewhat smaller mechanical reinforcement, but it more strongly increased processing viscosity due to its high specific surface area, which generated highly stable physical crosslinking that persisted even at processing temperatures of *T* ≥ 120 °C. Efficient matrix–filler interactions were confirmed by thermogravimetric analysis, where the better-exfoliated LAP generated a higher stabilization. The combination of strong mechanical reinforcement with outstanding gas-barrier properties makes the TPS/MMT and TPS/LAP nanocomposites attractive for food-packaging applications, where their natural origin, non-toxicity, bio-degradability, and abundance of nanocomposite components are an additional bonus.

## 1. Introduction

The increasing worldwide accumulation of highly resistant, and hence persistent, plastic waste has inspired a growing interest in research into biodegradable polymers, which would be able to decay similarly to the decay observed for natural biological waste [1]. Among the wide range of biopolymers currently being studied, starch is one of the most promising, due to its availability, low cost, high annual production through the growth of various crops, and the complete biodegradability of this plant material in soil, as well as in aqueous environments [2]. Neat (and dry) natural starch is infusible due to its extremely high molecular weight combined with its strong hydrogen bonding. However, with the help of plasticizers like glycerol, dispersed by subjecting the starting mixture to heat and shear [3,4,5], natural starch can be turned into thermoplastic starch (TPS) and further processed using conventional polymer technologies, such as extrusion and injection molding.

Despite these benefits, TPS still has significant disadvantages, such as high sensitivity to moisture (changes in properties due to water absorption or drying), limited mechanical strength, and time-dependent property changes (physical aging independent of moisture) [6]. Extensive efforts have been undertaken to mitigate the drawbacks of TPS, and have often aimed at reinforcing TPS with suitable fillers in order to obtain composites that meet the requirements for packaging applications, but can also be used in biomedicine (due to the biological origin and biocompatibility of starch), and for other technological applications [7,8,9,10]. The fillers most frequently studied in TPS matrices include cellulose microfibrils, nanofibers, and nanocrystals [11], as well as layered silicates (particularly clays) [12,13], metal oxides and their nanoparticles [14,15], and also carbonaceous materials [16,17], such as carbon nanoparticles, graphite, carbon black, and carbon nanotubes.

Layered silicate nano-clays, such as natural or organically modified montmorillonite (MMT) [18,19,20], have received considerable attention in research into TPS nanocomposites due to these clays’ high surface areas, cation-exchange capacities, non-toxicity, and natural abundance. However, notable improvements in TPS’s properties are achieved only if the clay platelets are at least intercalated or, ideally, fully exfoliated within the polymer matrix [21,22,23,24]. Dispersed nano-clays not only influence the mechanical and structural properties of TPS films, but they also significantly improve their barrier properties, thus reducing their water and oxygen permeability. This latter property is highly desirable for food and pharmaceutical packaging [20,25,26,27].

Synthetic nano-clays such as laponite RDS (LAP; a disk-shaped hectorite analog with platelet diameter of ca. 25 nm and platelet thickness of ca. 1 nm [28]) are known to act as strongly reinforcing, or even cross-linking agents in various polymer systems [29,30]. The mentioned LAP undergoes complete exfoliation if dispersed in water, and for this reason, it has attracted substantial interest as a reinforcing [31,32] or stabilizing [33] agent in numerous aqueous polymeric matrices.

While TPS/MMT systems have been thoroughly studied, LAP-reinforced TPS has not received a similar interest: only a few works dedicated to TPS/LAP systems have been published [34,35,36]. These studies have typically employed a relatively narrow range of LAP concentrations, usually from 0 to 5 wt.% [34], and occasionally up to 10 wt.% [35]. The visible clustering and agglomeration of clay platelets have been reported at LAP contents of around 10 wt.% [36]. Studies directly comparing LAP and MMT as reinforcing phases in TPS are especially rare, and to the best of our knowledge, only one has been conducted so far [35].

An additional knowledge gap concerns the procedure of preparing TPS-based nanocomposites. Previous studies performed by Ostafińska et al. [37], Ujcic et al. [38], and one that was recently extended by Kouka et al. [39] have demonstrated that a two-step protocol, involving solution casting (SC) followed by melt mixing (MM), yields exceptionally homogeneous TPS matrices (no heterogeneities in the TPS itself) and promotes a highly efficient nanofiller dispersion. Nevertheless, this advanced SC+MM processing technique has never been used for TPS/LAP or TPC/MMT systems. The other authors, who employed simpler and/or different preparation protocols for TPS/LAP or TPS/MMT nanocomposites, achieved moderate improvements in TPS’s properties [23,35,36,40,41,42]. Hence, a comparative study of this topic is of great interest.

In this work, we applied the SC+MM approach to the preparation of TPS/LAP and TPS/MMT nanocomposites for the first time, and we examined how LAP and MMT differ in their impact on the morphology, matrix–clay interactions, thermomechanical properties, and rheological behavior of the resulting nanocomposites. Another novel aspect of this work lies in its evaluation of a very broad filler concentration range, up to 15 wt.%, markedly exceeding the range examined in previously studied clay-based TPS systems. This was made possible by the novel SC+MM method, which efficiently prevented LAP agglomeration, even at 15 wt.%, and which also made a fairly good exfoliation and dispersion of the less attractive ‘simple’ MMT clay nanofiller possible. A considerable improvement in the mechanical properties of the novel nanocomposites was proven in both the microscale (indentation) and macroscale (DMTA and tensile tests). The studied products might be of interest as biodegradable packaging materials, while the novel preparation method, as well as the analysis of structure–property relationships, might be inspiring for the development of new nanocomposites with layered fillers.

## 2. Materials and Methods

### 2.1. Materials

Wheat starch (A-type) was supplied by Škrobárny Pelhřimov a.s. (Pelhřimov, Czech Republic). Thermoplastic starch (TPS) was prepared from wheat starch, as described below in Section 2.2. The first nano-clay filler, Laponite RDS (LAP), was supplied by BYK Additives & Instruments (Wesel, Germany). The second, organo-modified nano-clay filler Montmorillonite (MMT), Cloisite 15A, modified with dialkyldimethylammonium chloride (95-meq/100 g), was obtained from Southern Clay Products, Inc. (Gonzales, TX, USA). Anhydrous glycerol (C_3_H_8_O_3_; >99%) and sodium bromide (NaBr; >99%) reagents were bought from Lach-Ner s.r.o. (Neratovice, Czech Republic).

### 2.2. Preparation of Thermoplastic Starch and Its Composites

Thermoplastic starch (TPS) and its composites (TPS/LAP and TPS/MMT) were prepared by a two-step preparation protocol that consisted of solution casting (SC) followed by melt mixing (MM). The protocol has been described in our previous studies [37,38,39,43]. The slightly modified TPS preparation that was employed in this study is briefly described in Section 2.2.1 and Section 2.2.2. The list of all prepared composites is given in Table 1.

#### 2.2.1. Solution Casting

The powdered nanofiller (LAP or MMT) was dispersed in water so that the final weight fraction corresponded to 0, 1, 5, 10, or 15 wt.% of the filler. The starch powder (70 wt.%) was premixed with glycerol (30 wt.%) and the water suspension of the filler (6 parts of water with filler per 1 part of starch). The premixing was performed in a beaker with a magnetic stirrer for 30 min at room temperature. The premixed suspension was transferred to a stronger mechanical stirrer (RZR 2020, Heidolph, Heidolph Scientific Products GmbH, Schwabach, Germany) combined with a heating bath, where the temperature was elevated to 70 °C (temperature ramp ~5 °C/min). When the temperature was approaching 70 °C, the starch started to gelatinize. The beginning of the gelatinization was accompanied by a significant increase in the suspension’s viscosity. The composition was mixed for another 15 min while keeping the temperature at 70 ± 3 °C. Approximately halfway through the mixing time, 5 mL of deionized water was added to the suspension to decrease the viscosity slightly, which improved the mixing. After 15 min at 70 °C, the solution was cast onto a polyethylene (PE) foil to form a film (ca 2 mm thick). The thin film was left to dry at room temperature for three days to allow for the evaporation of residual water.

#### 2.2.2. Melt Mixing

The solution-cast and dried TPS and composites were processed by melt mixing using a twin-screw laboratory mixer (Brabender Plasti-Corder, Duisburg, Germany). The melt mixing was shown to further increase the homogeneity of the TPS-based systems [37,39,44]. The samples were mixed in the chamber, which was pre-heated to the nominal processing temperature of 120 °C, using a rotation speed of 60 rpm for 8 min, while recording the real processing temperature and torque. The mixed samples were compression-molded into plaques with a thickness of 2 mm, using a laboratory hot press (Fontijne Grotnes; Vlaardingen, The Netherlands). The compression molding was performed in three sub-steps: (i) a pressure of 50 kN was applied at a temperature of 130 °C for 2 min to deaerate, (ii) a of pressure 100 kN was applied at the same temperature, 130 °C, for another 2 min, and (iii) cooling to room temperature using water while maintaining a pressure of 100 kN (cooling ca. 10 min, cooling rate ca. 10 °C/min).

#### 2.2.3. Storage of Prepared Samples

The final TPS plaques were stored at room temperature (ca 20 °C) in a desiccator over a supersaturated solution of sodium bromide (relative humidity = 57%). The samples were kept in the desiccator at all times and were removed only for characterization measurements, as described below. As the measurement times were reasonably short for all employed methods, the influence of the surrounding environment can be assumed to be minimal.

### 2.3. Morphology Characterization

#### 2.3.1. Scanning Electron Microscopy

Sample morphology was studied by scanning electron microscopy (SEM) using a microscope MAIA3 (Tescan, Brno, Czech Republic). Sample morphology and the interfacial adhesion of fillers was studied from the fracture surfaces. The fracture surfaces were prepared by breaking the samples while they were submerged in liquid nitrogen [45]. The fractured specimens were fixed on metallic supports with a conductive silver paste (Leitsilber G302; Christine Groepl, Tulln, Austria), sputter-coated with a thin platinum layer (vacuum sputter coater SCD 050; Leica, Austria; thickness of the Pt layer: approx. 4 nm), and observed by means of secondary electron imaging (SEM/SE) and back-scatter electron (SEM/BSE) imaging at accelerating voltage 3 kV. Before the SEM analysis, the samples were placed for 24 h in a dry desiccator (with silica gel), which improved their stability during the vacuum sputter-coating process (minimization of artifact cracks on the surface).

#### 2.3.2. Transmission Electron Microscopy

The filler’s dispersion in the starch matrices was studied by transmission electron microscopy (TEM). Ultrathin sections (approximately 60 nm thick) were cut using an ultramicrotome (Ultracut UTC, Leica, Vienna, Austria). The slices were put on supporting Cu grids and observed with the Tecnai G2 Spirit Twin 12 microscope (FEI, Brno, Czech Republic) using the bright field imaging mode at an accelerating voltage of 120 kV.

#### 2.3.3. Wide-Angle X-Ray Scattering

Wide-Angle X-ray Scattering (WAXS) patterns were collected using a high-resolution Anton Paar XRDynamic 500 diffractometer (Graz, Austria) with an Advacam pixel detector (Prague, Czech Republic), utilizing parallel beam geometry under CuKα radiation (wavelength λ = 1.54 Ǻ) in the 2θ range of 3–80° with a step of 0.01° and 30 s measurement time at each step. The degree of crystallinity was determined using XRDanalysis PRO software (Anton Paar, Graz, Austria, version 1.2.1.4281) by calculating the ratio of the crystalline peak area to the total area under the WAXS curve.

### 2.4. Micromechanical Properties

Micromechanical properties were measured with a microindentation hardness tester (MCT tester; CSM, Switzerland). The instrumented microindentation hardness testing (MHI) experiments were performed using a Vickers tip (a diamond square pyramid with an angle between non-adjacent faces of 136°), which was forced against the flat surface of a specimen. The flat, smooth-cut surfaces for testing were prepared with a rotary microtome (RM 2255, Leica, Vienna, Austria). As described above, the samples were stored at room temperature in a desiccator above a supersaturated solution of NaBr, which yielded a relative humidity of 57%; in order to achieve maximum reproducibility, the samples were left to equilibrate in the desiccator for one week before the MHI measurements. The micromechanical properties were calculated from the *F*-*h* curves, i.e., from the loading force, *F*, which was measured as a function of tip penetration depth, *h*. For each sample, three independent cut surfaces were prepared. For each measured surface, at least 10 independent measurements/indentations were made, and the final results were averaged. The parameters of MHI measurements were as follows: maximal loading force *F*_max_ = 500 mN, dwell time (time of maximal load) 60 s, and linear loading and unloading rates 15,000 mN/min (i.e., ca. ~2 s to achieve and release *F*_max_). The evaluated micromechanical properties were as follows: indentation modulus (*E*_IT_; proportional to macroscopic elastic modulus), indentation hardness (*H*_IT_; proportional to macroscopic yield stress), Martens hardness (*H*_M_; also referred as universal hardness, proportional to macroscopic yield stress as well), indentation creep (*C*_IT_; related to the macroscopic creep), and elastic part of the indentation work (*η*_IT_; defined as ratio of elastic deformation to total deformation). The calculations of *E*_IT_ and *H*_IT_ are based on the theory by Oliver and Pharr [46], while the values of *H*_M_, *C*_IT,_ and *η*_IT_ are independent of the O&P theory [47]. The exact definitions of the above-listed micromechanical properties can be found in textbooks on micromechanical properties [48,49] and a more detailed description of the MHI measurements is also in our recent studies [50,51,52].

### 2.5. Macromechanical Properties

The tensile tests were carried-out on an ARES-G2 rheometer (TA Instruments, New Castle, DE, USA) at 22 °C. The “Axial” measurement method was used, with a specimen gauge length of 4 mm and a cross-head speed of 0.0667 mm·s^−1^ (≡1.667%·s^−1^), which produced the same relative deformation rate (in %·s^−1^) as that of an ISO 37/4 specimen (10 mm gauge length) stretched at a 10 mm/min rate. Sample dimensions: overall specimen length, 20 mm; gauge length (between sample holders), 4 mm; width, 2 mm; thickness, 1 mm. For each material, no less than three measurements were performed, and representative tensile curves closest to the average response are presented.

### 2.6. Thermomechanical Properties and Rheology

#### 2.6.1. Thermomechanical Properties

The thermomechanical properties of TPS samples were analyzed using dynamic mechanical thermal analysis (DMTA). Tests were performed on rectangular specimens with an ARES G2 rheometer (TA Instruments, New Castle, DE, USA) operating in an oscillatory regime at 1 Hz. The deformation amplitude ranged from 0.01 to 3%, adjusted automatically by the auto-strain function, which was sensitive to the sample stiffness. The temperature range studied was from −90 to 120 °C, with a heating rate of 3 °C/min. The temperature-dependent curves of the storage shear modulus (G′), loss modulus (G″), and loss factor tan (δ) were recorded. The standard specimen dimensions were 40 mm × 10 mm × 2 mm.

#### 2.6.2. Rheology

The rheological properties of thermoplastic wheat starch (TPS) filled with either MMT or Laponite RDS at 120 °C were evaluated using an ARES G2 rheometer in controlled deformation mode (TA Instruments, New Castle, DE, USA). The experiments were performed using parallel plate geometry (30 mm diameter) featuring cross-patterned surfaces to prevent sample slippage. The experimental regime was oscillatory, with a frequency of 1 Hz. The sample thickness was 2 mm. As a first step, the linear viscoelastic range (LVER) was determined based on the relationship between the storage modulus and strain amplitude. Subsequently, frequency sweep tests were conducted over a range of 0.1 to 100 rad/s at a constant oscillatory deformation amplitude of 0.05% (within the established LVER). To ensure thermal equilibrium, all samples were conditioned at 120 °C for 2 min prior to each measurement. Each frequency sweep was carried out twice for every sample.

### 2.7. Thermogravimetric Analysis

TGA was carried out on a Pyris 1 TGA thermogravimetric analyzer (Perkin Elmer, Waltham, MA, USA) at a temperature ranging from 35 to 850 °C, at a heating rate of 10 °C/min, while the gas flow was held at 20.0 mL/min. The TGA analyses were carried out in nitrogen, and also in air.

### 2.8. Gas Barrier Properties

The most promising TPS nanocomposite sample was subjected to a characterization of its gas transport properties at 30 °C. Films of a thickness of ca. 180 µm were prepared for this purpose. The characterization was performed by a high-vacuum apparatus equipped with a static permeation cell [53]. After high vacuum was achieved, a given gas (oxygen, hydrogen, carbon dioxide, or water vapor) was conducted into the feed part of the cell at a constant pressure *p_i_* (which was greater than the atmospheric pressure). The permeability of the respective gas was evaluated from the measured increase in pressure Δ*p*_p_ that occurred in the time interval Δ*t* in the calibrated volume *V_p_* of the cell. The following formula was used:

$$P=\frac{\Delta p_{p}}{\Delta t}\cdot \frac{V_{p\;}l}{Ap_{i}}\cdot \frac{1}{RT}$$

where *A* is the area, *l* is the film thickness, *R* is the gas constant, and *T* is the temperature. From the film thickness *l* and from the observed time lag *Θ*, the diffusion coefficient *D* for the corresponding gas was obtained:

$$D=\frac{l^{2}}{6\theta }$$

The solubility coefficient (*S*) of the given gas was determined as the ratio of the above-determined permeability (*P*) and diffusion coefficient *D*:

$$S=\frac{P}{D}$$

### 2.9. Processing Properties

During the melt mixing of TPS in a laboratory kneader (Section 2.2.2), we recorded the values of *torque* (TQ; in N/m) and *real processing temperature* (*T*_p_; in °C) as a function of processing time (t; in seconds). The *torque* is the rotational equivalent of the linear force that is needed to mix the melt in the kneader chamber. The *real processing temperature* is almost always higher than the *nominal processing temperature* (i.e., the temperature to which the kneader chamber is pre-heated before the mixing) due to the internal friction of the melt. To achieve maximal reproducibility, the experimental chamber of the kneader was always filled with the same amount of material (75 g) at the same loading speed (the chamber was fully loaded in 2 min). Moreover, the final values of TQ and *T*_p_ were evaluated at the final part of the TQ-*t* and *T*-*t* curves, i.e., after ca. 6 min, when the curves reached a plateau, indicating that the mixing had achieved a steady state.

## 3. Results and Discussion

### 3.1. Morphology

**Figure 1:**
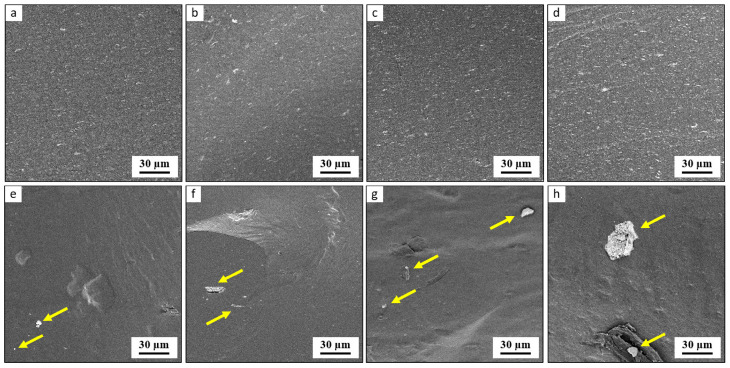
SEM/BSE micrographs of fracture surfaces of TPS/LAP (**a**–**d**) and TPS/MMT (**e**–**h**) systems. The concentration of the filler increases from left to right: (**a**,**e**) = 1 wt.%, (**b**,**f**) 5 wt.%, (**c**,**g**) 10 wt.%, and (**d**,**h**) = 15 wt.%. The yellow arrows mark MMT agglomerates.

**Figure 2:**
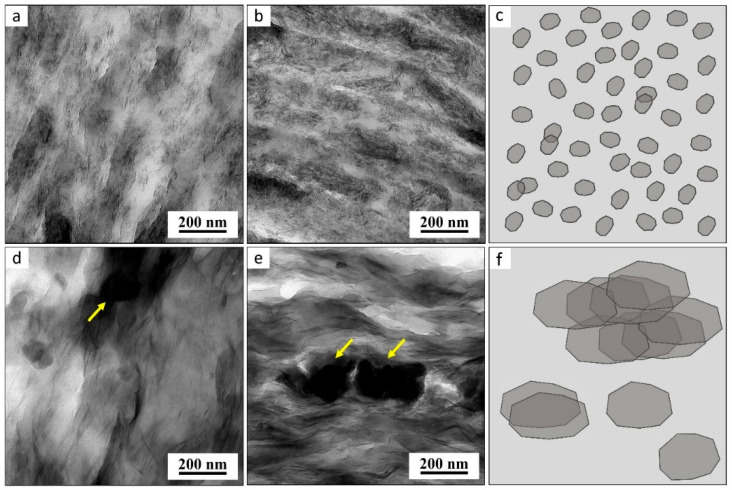
TEM micrographs and schemes of selected TPS/LAP and TPS/MMT composites: (**a**) TPS/LAP-10, (**b**) TPS/LAP-15, (**c**) scheme of LAP’s dispersion in the TPS matrix, (**d**) TPS/MMT-10, (**e**) TPS/MMT-15, and (**f**) scheme of MMT’s dispersion in the TPS matrix. In the TEM bright field imaging, the LAP and MMT sheets appear as darker objects in the brighter polymer matrix. The micrographs and schemes document the less homogeneous dispersion, overlapping, and stacking of MMT platelets in comparison with the finely dispersed LAP platelets.

**Figure 3:**
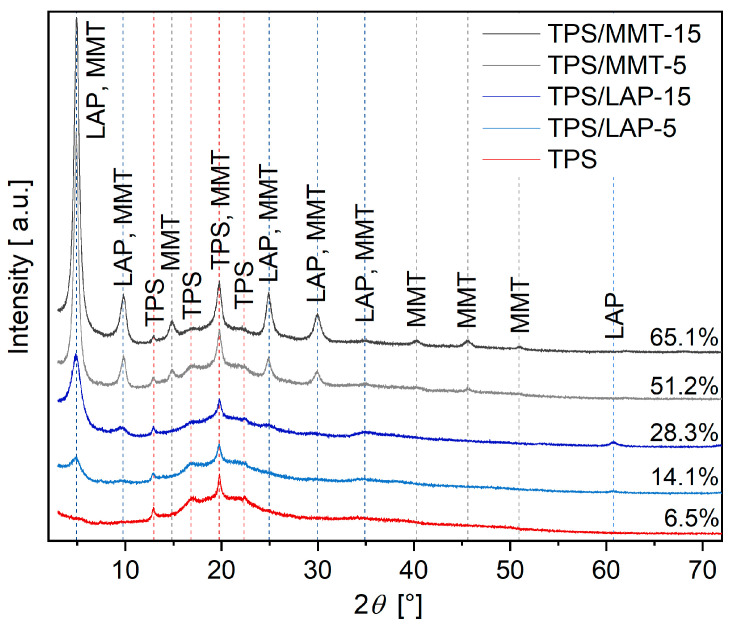
WAXS diffraction patterns and crystallinities of selected samples: pure TPS and TPS with 5% and 15% of LAP and MMT fillers. The crystallinity values are located at the right side of each diffraction pattern.

The morphology of the TPS/MMT and TPS/LAP composites was studied by SEM, TEM, and WAXS. SEM imaging with backscattered electrons (SEM/BSE; Figure 1) showed the overall dispersion of the nanofillers in the TPS matrix. The higher-magnification TEM micrographs obtained by means of bright field imaging (TEM/BF; Figure 2) showed the dispersion of the individual MMT and LAP nanoplatelets in the polymer. WAXS diffractograms (Figure 3) illustrated the extent of the exfoliation of the MMT and LAP nanoplatelets along with the residual crystallinity of the TPS matrix.

Figure 1 shows the representative SEM/BSE micrographs of the fracture surfaces of all of the TPS nanocomposites investigated. In SEM/BSE imaging, the possible agglomerates appear as white spots due to the higher average atomic number of the inorganic filler (LAP or MMT) in comparison with the polymer matrix (TPS) [45]. The TPS/LAP nanocomposites (Figure 1, upper row) exhibited a very fine dispersion of the filler up to 15 wt.%, with only occasional small agglomerates (small white dots in SEM/BSE). Increasing the concentration of LAP led to an increasing number of small agglomerates in the TPS matrix. In contrast, the TPS/MMT nanocomposites (Figure 1, lower row) showed a more heterogeneous distribution of nanofiller. Most of the MMT seemed to be well dispersed in the matrix, like in the case of the TPS/LAP composites, but occasional larger agglomerates of MMT (bigger white spots, marked with yellow arrows in Figure 1e–h), could be observed as well.

Figure 2 displays the TEM/BF micrographs of ultrathin sections of selected TPS/LAP and TPS/MMT composites. Previous studies have reported that the synthetic nano-clay LAP can be easily dispersed in water, and that it is ideally suited for obtaining homogeneous clay platelet dispersions with loadings up to 10 wt.% in various polymer matrices [54,55]. In this work, we achieved well dispersed LAP nanoplatelets not only in the TPS/LAP-10 composite (Figure 2a), but also in the heavily filled TPS/LAP-15 composite (Figure 2b). The dispersion of the filler was also fine in the samples of TPS/MMT-10 (Figure 2c) and TPS/MMT-15 (Figure 2d), although occasional larger agglomerates could be observed (yellow arrows in Figure 2c,d). This good dispersion and exfoliation in both TPS/LAP and TPS/MMT composites, even at high filler loadings of 10–15 wt.%, is a remarkable success in view of the previous literature about MMT dispersion in starches [23,26,34,35]. The novel two-step preparation protocol employed in the present work, as well as the high melt viscosity of the TPS during this process, appear to have been the main success factors. Among the previous works about TPS/MMT composites, except in [26], no TEM images of the achieved dispersion were shown, and the TEM analysis in [26] indicated that at 6 wt.% of MMT (maximum tested loading), a considerable concentration of the filler in larger nanoaggregates had already occurred, combined with a fairly diluted fraction of exfoliated nanoplatelets, whose concentration was somewhat oscillating. In [23,34] (no TEM), the maximum tested MMT concentration was 5 wt.%. In [35] the authors incorporated up to 10% of different clays (including MMT) into TPS, but no TEM was shown. Besides the fairly good dispersion of MMT, Figure 2c,d also visualizes the approximate MMT platelet size, ca. 100–180 nm (for comparison: LAP in Figure 2a,b is ca. 30 nm). Especially at 15 wt.%, the dispersed platelets appear to be overlapping to a considerable degree (transparent but darker regions in Figure 2d, similar to schematic representation of the overlaps in Figure 2e). These partly overlapped and multi-layered nanofiller structures, which extend over the whole material, appear to have played an important role in the attractive mechanical and gas barrier properties of TPS/MMT-15.

Figure 3 compares the WAXS diffraction patterns of the selected TPS/LAP and TPS/MMT composites. The crystallinity of the composites is significantly higher than that of pure TPS, particularly for TPS/MMT. This increase is mainly due to the clay nanofillers, while the peaks associated with TPS crystallinity remain largely unchanged. In the TPS/MMT composites, the nanofiller-related reflections are regularly spaced with a step of approximately 4.93° up to the 10th order, indicating an ordered lamellar structure with a lamellar spacing of ~1.79 nm and a coherence length of ~10.3 nm, as estimated using the Scherrer formula. The multi-layered, stacked, and platelet-overlayed structures, which were observed (Figure 2) and confirmed by WAXS (Figure 3, gray lines), were postulated to play a considerable role in the mechanical properties of the TPS/MMT composites, as discussed below in more detail. In TPS/LAP composites, many of the higher-order reflections are absent, consistent with the considerably high degree of exfoliation of the LAP platelets observed by SEM (Figure 1) and TEM (Figure 2).

### 3.2. Micromechanical Properties

**Figure 4:**
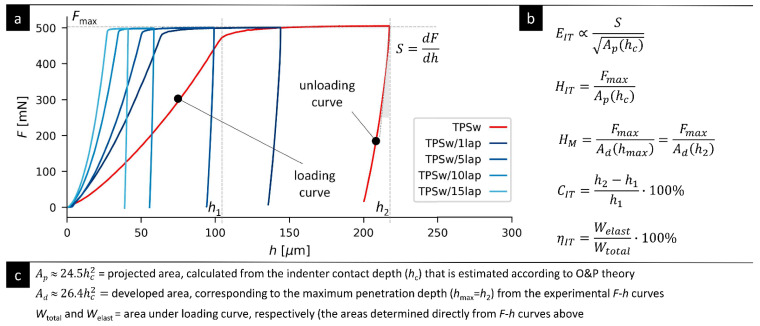
Raw data and principles of the instrumented microindentation hardness testing (MHI) experiments: (**a**) Representative *F*-*h* curves, where *F* is the loading force, and *h* is the penetration depth of the indenter tip into the investigated material; the *F*-*h* curves show the stiffening of the TPS/LAP composites with the increasing concentration of the filler. (**b**) Definition of the basic micromechanical properties that were calculated from the experimental *F*-*h* curves; the quantities are (from top to bottom) the indentation modulus, *E*_IT_; indentation hardness, *H*_IT_; indentation creep, *C*_IT_; and elastic part of the indentation work, *η*_IT_. (**c**) Additional definitions of auxiliary quantities that are used in the MHI calculations.

The micromechanical properties of all of the prepared TPS composites were assessed from instrumented microindentation hardness testing (MHI). The experimental details of the MHI measurements are given in Section 2.4. The theoretical background on MHI data collection and interpretation can be found in relevant textbooks [49,56] and the references therein. The principles of the MHI measurements and data evaluation are given in Figure 4; more details about the micromechanical properties of polymer systems can be found in our previous studies [47,50,51].

Figure 4 shows the raw data from the MHI measurements and the *F*-*h* curves (where *F* is the loading force and *h* is the penetration depth of the tip into the investigated sample) together with basic definitions of the evaluated micromechanical properties (*E*_IT_, *H*_IT_, *C*_IT_, and *η*_IT_; see also Section 2.4). The *F*-*h* curves in Figure 4 document that the micromechanical properties of the prepared TPS/LAP composites changed consistently: for the same loading force, *F*, the penetration depth, *h*, decreased with an increasing concentration of nanofiller. Similar trends were observed for the TPS/MMT composites, as discussed below.

**Figure 5:**
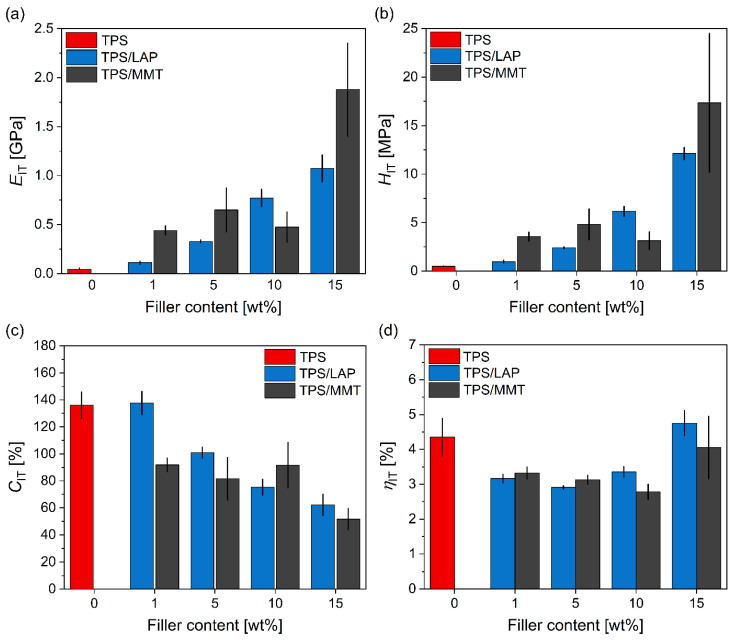
Stiffness-related properties of TPS and its composites after SC+MM, obtained from microindentation hardness testing: (**a**) indentation modulus, *E*_IT_; (**b**) indentation hardness, *H*_IT_; (**c**) indentation creep, *C*_IT_; and (**d**) *η*_IT_ elastic part of indentation work. For each sample, at least 90 indentations were performed (30 indentations in 3 independent cut surfaces per sample), and the results were averaged; the error bars represent estimated standard deviations.

Figure 5 summarizes the final micromechanical properties of all of the studied systems: indentation modulus (*E*_IT_, Figure 5a), indentation hardness (*H*_IT_, Figure 5b), indentation creep (*C*_IT_, Figure 5c), and the elastic part of the indentation work (*η*_IT_, Figure 5d). We note that the micromechanical properties are related to macroscale properties: *E*_IT_ is proportional to macroscale elastic modulus (*E*_IT_ ∝ *E*; according to Oliver & Pharr’s theory [57]), *H*_IT_ is proportional to macroscale yield strength (*H*_IT_ ≈ 3*Y*; according to the Tabor relation [58]), *C*_IT_ was shown to be related to macroscale creep [59], and *η*_IT_ is connected with the overall elasticity of the sample [47,49].

In the case of the TPS/LAP and TPS/MMT composites, the increasing concentration of the nanofiller resulted in an increase in *E*_IT_ and *H*_IT_ (higher stiffness and hardness of the filled systems), a decrease in *C*_IT_ (higher resistance of the filled systems to long-term deformation), and a slight increase in *η*_IT_ (higher elasticity connected with the lower plastic deformation and creep of the filled systems). The increase in the stiffness-related properties (*E*_IT_, *H*_IT_, and their macroscale analogs) with the concentration of inorganic filler is a general trend observed in numerous studies [44,60], while the behavior of the viscosity-related properties (*C*_IT_, *η*_IT_, and their macroscale analogs) depends on the polymer matrix [44,61].

**Figure 6:**
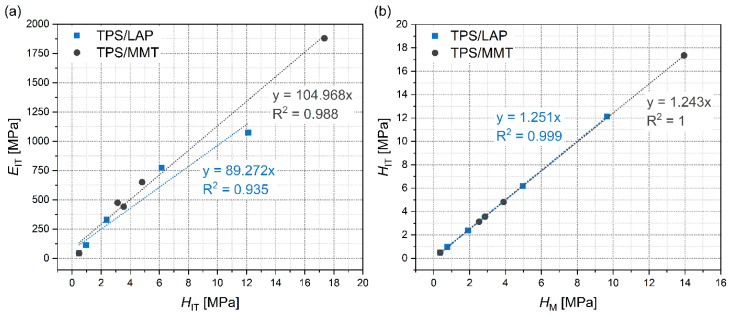
The correlations between stiffness-related micromechanical properties: (**a**) *E*_IT_-*H*_IT_ and (**b**) *H*_IT_-*H*_M_. The linear relations among the three quantities are in agreement with theoretical predictions [49].

Figure 6 documents that the stiffness-related micromechanical properties follow the theoretically predicted trends, which confirms the reliability and reproducibility of our microscale measurements. Figure 6a displays the linear correlation between *E*_IT_ and *H*_IT_. This is based on the detailed study by Struik [62], who found an approximate relation between the macroscale tensile modulus (*E*) and macroscale tensile yield stress (*Y*) of amorphous polymers (*E* ≈ 30*Y*). Later, it was shown that Struik’s relation also holds for semicrystalline polymers, polymer blends, and polymer composites, although the proportionality constant may vary and differ from system to system [3,51]. By combining Struik’s relation (*E* ≈ 30*Y*) with the above-mentioned O&P theory (*E*_IT_ ≈ *E*; [57]) and Tabor’s relation (*H*_IT_ ≈ 3*Y*; [58]), we get the theoretically predicted, approximate linear correlation between the indentation modulus and indentation hardness (*E*_IT_ ≈ 10*H*_IT_). In our case (Figure 6a), the proportionality constant is considerably higher than the approximate value of 10 due to the fact that TPS is a rather soft polymer (low *H*_IT_), but the linear correlation holds very well. Figure 6b shows the linear correlation between the indentation hardness (*H*_IT_) and Martens hardness (*H*_M_). The *E*_IT_ and *H*_IT_ values are calculated from the *F*-*h* curves according to the O&P theory, while the *H*_M_ values are calculated directly from the maximum penetration depth (*h*_2_ in Figure 4). The fact that the *H*_IT_ and *H*_M_ values are proportional indicates that the O&P theory is a reasonable approximation for the prepared TPS composites and that the calculated *E*_IT_ and *H*_IT_ values are relevant. This was further confirmed by the strong correlation between the microscale *E*_IT_ moduli and macroscale |G*| moduli from the DMTA measurements, as discussed below.

### 3.3. Macromechanical Properties

**Figure 7:**
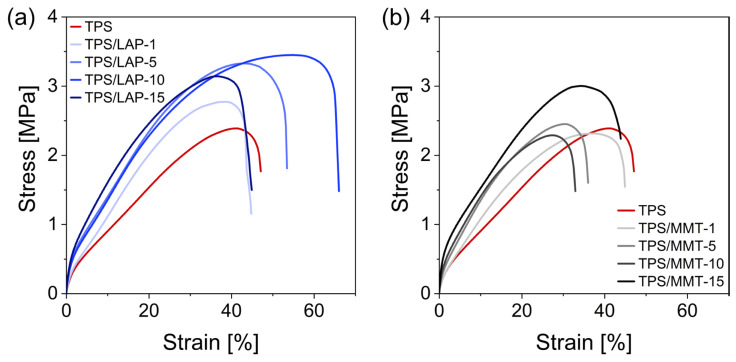
Stress–strain curves of TPS nanocomposites from tensile testing experiments: (**a**) TPS/LAP (blue lines) and (**b**) TPS/MMT (gray lines); the tensile curve of the neat matrix (TPS; red line) is shown in both diagrams as a reference.

The macromechanical properties of the studied nanocomposites at room temperature, especially their extensibility and toughness, were evaluated by means of tensile testing (Figure 7). The stress–strain curves generally indicate a good dispersion of both nanofillers: the extensibility does not deteriorate and is even improved in many cases, which indicates the absence of serious morphological defects that would support crack formation and propagation. The toughness was also often improved by the clays. This can be attributed to strong sacrificial physical crosslinks between the matrix and filler that generate mechanical resistance, and the disruption and recombination of which during large deformations acts as an efficient energy-dissipation mechanism. These interactions are additionally documented by the increase in the slopes of the tensile curves.

In the case of the LAP filler, the extensibility increases with 5% of this clay (visibly) and with 10% (vastly), while at 15% it strongly recedes to a value slightly smaller than that observed for the neat matrix. Nevertheless, the toughness at 15% LAP (area under the curve) is still distinctly higher than in the case of neat TPS. The drop in extensibility when increasing from 10 to 15% LAP can be partly attributed to the slightly more frequent observation of LAP aggregates at a loading of 15% (see Section 3.1), which (as stiff heterogeneities) might support crack initiation, in combination with the marked rigidification of the TPS matrix at 15% LAP loading (as observed by DMTA and discussed further below). This rigidification effectively generates a semi-glassy state (easy crack propagation). The starch-rich phases of TPS that effectively control the extensibility (see DMTA discussion), and which interpenetrate the whole sample, are still in a glassy state at the temperature at which the tensile tests are performed (25 °C) and are strongly reinforced by the LAP nanoplatelets (see DMTA discussion).

In the case of the less-perfectly exfoliated MMT, the extensibility increases slightly at first (1%), then visibly drops at 5 and 10% (down to similar values in both cases). But finally, when going from 10 to 15% MMT, the extensibility markedly increases again, up to ca. the same value as the neat TPS. Additionally, the toughness of the TPS/MMT-15 system is distinctly higher than that of the neat matrix. The trends for the MMT-filled systems seem to indicate that the filler’s dispersion might be more uneven at low loadings, while at 15%, the high amount of MMT enforces a fairly even dispersion of the large MMT platelets during the nanocomposite preparation. TPS/MMT-15 does not surpass the extensibility of TPS, most likely due to the combined effects of heterogeneities (larger MMT aggregates occasionally observed by electron microscopy) and of the marked TPS rigidification. The aforementioned micromechanical tests, as well as the following DMTA analyses, also indicate a tremendous improvement in mechanical properties when going from 10 to 15% of MMT.

### 3.4. Thermomechanical Properties

**Figure 8:**
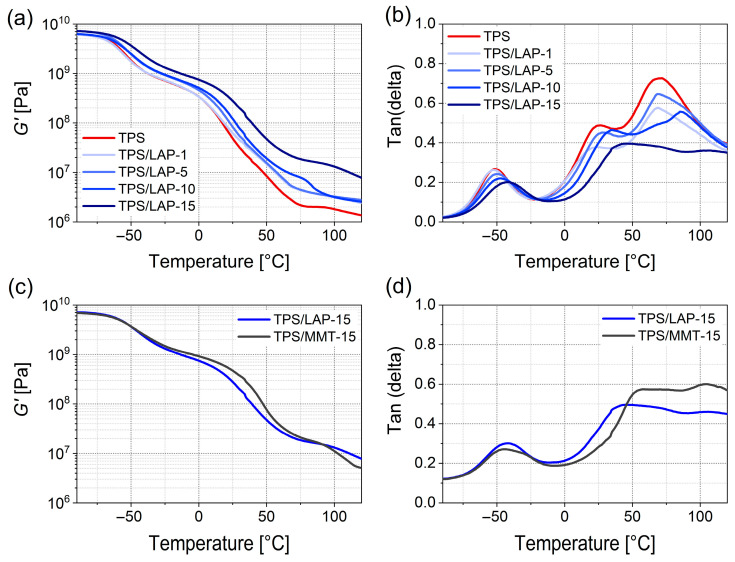
Thermomechanical properties of the studied TPS/clay nanocomposites, measured by DMTA in rectangular torsion mode, in the temperature range from −90 to +120 °C: (**a**) storage modulus *G*′ = f(*T*) of TPS/LAP nanocomposites containing 1, 5, 10, and 15 wt.% of the filler, as well as of neat TPS; (**b**) loss factor tan(δ) = f(*T*) of the same set of samples. The last two figures (**c**,**d**) compare LAP-based and MMT-based nanocomposites with 15 wt.% of the filler: (**c**) *G*′ = f(*T*); (**d**) tan(δ) = f(*T*). The DMTA characteristics of the TPS/MMT nanocomposites and their comparisons with TPS/LAP composites for the filler loadings of 1, 5, and 10% are shown in Figures S1 and S2, respectively, in the Supplementary Information File.

The studied TPS/LAP and TPS/MMT nanocomposites were strongly reinforced by both clay fillers, which is illustrated by the thermomechanical (DMTA) analyses presented in Figure 8 (as well as in Figures S1 and S2 in the Supplementary Information). The DMTA profiles of all prepared materials were similar, as documented by the temperature dependence of the *G*′ (Figure 8a) and tan(δ) (Figure 8b) of all the LAP-based systems. The small differences between LAP- and MMT-based nanocomposites are illustrated in Figure 8c,d, with the example of the *G*′ = f(*T*) and tanδ = f(*T*) curves at a 15 wt.% loading of both fillers. The DMTA results obtained for the TPS/MMT systems, as well as the LAP vs. MMT comparisons for the loadings of 1, 5, and 10%, are shown in Figures S1 and S2.

The reinforcement offered by both nano-clays is especially strong at elevated temperatures of up to ca. 70 °C, as documented in Figure 8c. Interestingly, the less-perfectly exfoliated MMT clay systematically generates a somewhat stronger reinforcing effect at all loadings. In accord with the TEM and WAXS results (Figure 2 and Figure 3), an important role in this surprising finding can be assigned to the effect of its larger-sized and partly overlapping MMT platelets, including multi-layers, as observed by TEM and XRD. As discussed in detail in Section 3.7, the larger MMT platelets display a markedly higher aspect ratio and hence a higher tendency towards mechanical percolation. Additionally, their multi-layers should be mechanically stronger. However, the differences between the reinforcing effects of MMT and LAP nearly disappear in the DMTA transition region above 100 °C, and 15% LAP reinforces more strongly at 120 °C. Hence, at T > 100 °C, the effect of the higher effective specific surface area of the smaller LAP nanosheets dominates.

Three glass transitions can be observed in the prepared TPS systems, as documented in Figure 8 and Figure S1 in the Supplementary Information. According to the previous literature [37,38,43,63,64,65,66,67,68,69], up to four thermo-mechanical transitions can be observed in TPS. The first is the lowest glass transition of the amorphous, plasticizer-rich domains (*T*_g1_; usually between −60 and −20 °C; in our case, ca. −50 °C). The second is the intermediate glass transition corresponding to the amorphous, starch-rich domains (*T*_g2_; usually between 0 and 30 °C; in our case, near 25–30 °C). The third is the high-temperature glass transition of the starch-rich domains that contain remnants of semi-crystalline regions and/or amylose domains (*T*_g3_; usually between 30 and 100 °C; in our case, around 75 °C but approaching 110 °C at the highest loads of the fillers). The final thermo-mechanical transition is the ultimate melting (*T*_m_) of the bulk TPS, which is typically observed between 120 and 150 °C; the final Tm transition was outside of our measurement range (from −90 to 120 °C, see Section 2.6.1).

Four plateau-like thermal regions, separated by the above-mentioned three glass transitions (*T*_g1_–*T*_g3_), are observed in the *G*′ = f(*T*) plots in Figure 8a and Figure S1a. The first three plateau-like regions correspond to the following: (i) the stiff glassy state below *T*_g1_, (ii) the soft glassy state below *T*_g2_, and (iii) the stiff rubbery state below *T*_g3_. The fourth plateau region, corresponding to the soft rubbery state below *T*_m_, is partially visible after the third glass transition. This fourth plateau would be ended by the final melting, which was not visible in Figure 8 and Figure S1, as explained in the previous paragraph. Except for the stiff glassy state, where the effect of both fillers was minimal, in all remaining plateaus, both the LAP and MMT nanofillers influenced the TPS systems’ thermomechanical properties or even raised their moduli markedly. This implies that the nanofillers interacted strongly with all phases of TPS, i.e., with the plasticizer-rich (diluted) starch, with the amorphous starch-rich (plasticizer-deficient) phase, and with the semi-crystalline starch-rich phase.

Reinforcement indices (defined as RI = *G*′_(nanocomposite)_/*G*′_(TPS)_) yield interesting structure–property relationships (Figure 8a). In the soft glassy region (below *T*_g2_) and stiff rubbery region (below *T*_g3_), at least one constituent phase of the TPS matrix was still in the glassy state. Here, the larger-sized, less exfoliated, and partly overlapping MMT nanofiller (Figure 2) generated slightly higher RI values. In the soft glassy region (2nd plateau), we had RI(LAP) 2.2 < RI(MMT) ≈ 2.7, and in the stiff rubbery region (3rd plateau), we observed RI(LAP) ≈ 8.5 < RI(MMT) ≈ 9.3 (the RI values for 15% filler). In these *T*-regions, the strong physical crosslinking provided by the domains of TPS, which were still glassy, seemed to amplify the effect of the larger (and overlapping) filler MMT. In the last soft, rubbery region (above *T*_g3_), where all the phases of TPS exhibited segmental mobility, the almost-completely exfoliated and better-dispersed LAP nanofiller resulted in stronger reinforcement. The RI values increased with the nanofiller content in the following ranges: RI(LAP) ≈ 1.91–5.82 > RI(MMT) ≈ 1.65–3.7, which can be attributed to the effect of the higher specific surface area of LAP. Additional details are discussed in the Supplementary Information (Figure S2).

The shifts in the glass transition temperatures (*T*_g1_–*T*_g3_), which were caused by LAP and MMT, are illustrated by the corresponding peaks in the tanδ = f(*T*) curves in Figure 8b and Figure S1b. In general, the *T*_g_ values were up-shifted with increasing clay content, at least at higher filler loadings. This indicated the increasing immobilization of the respective TPS phases due to their interactions with the clay. In the TPS/LAP systems (Figure 8b), the intensity of the tan(δ) peaks of all *T*_g_s systematically decreased with rising LAP concentration. This is consistent with the more elastic character of the TPS/LAP composites caused by the interactions between the TPS matrix and the well-exfoliated LAP. In the TPS/MMT systems (Figure S1b), the *T*_g3_ peaks did not change with the increasing MMT loading until 10 wt.%. The *T*_g2_ peaks even increased in intensity as the MMT loading rose to 10%. Only the highest MMT loading of 15% caused a marked drop in the peaks of both *T*_g2_ and *T*_g3_. This could be explained by the friction in the MMT multi-layers and stacks. Only at the highest loading of 15 wt.%, when the MMT platelets formed a percolating and relatively stiff structure, did the elasticity of this structure outbalance the friction, and thus, tan(δ) dropped. On the other hand, the transition at *T*_g1_ displayed very simple trends that were similar to both LAP and MMT: an up-shift in *T*_g_ combined with a decrease in peak height, which can be attributed to the increasing immobilization combined with increasing physical crosslinking (elastic character) caused by both clays. This suggests that the exfoliation and dispersion of the LAP and MMT platelets in the plasticizer-rich (diluted) TPS phase were comparable. Some additional discussion of the DMTA results is provided in the Supplementary Information (SI).

**Figure 9:**
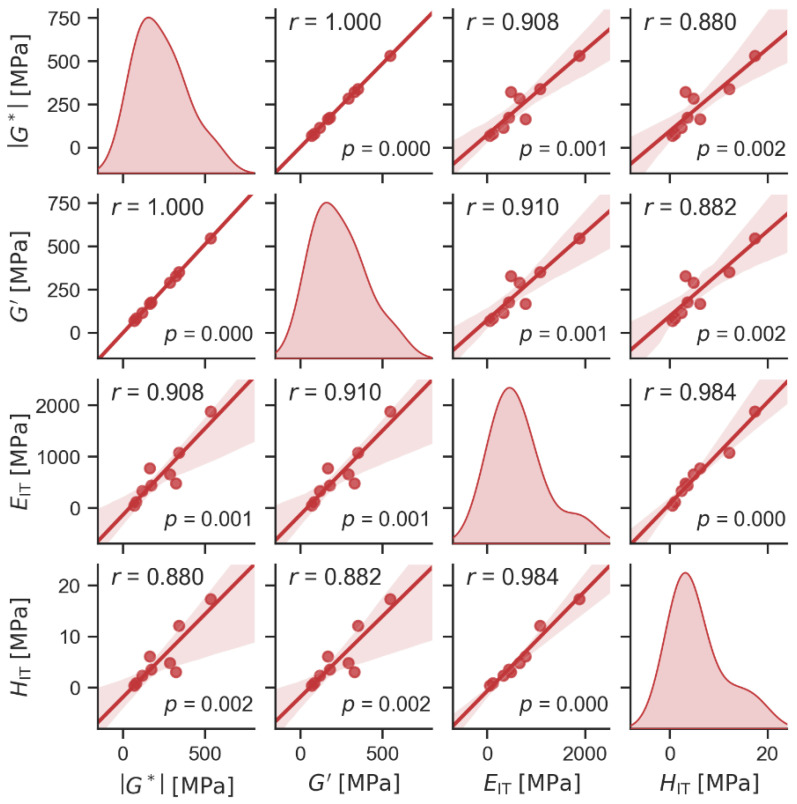
Scatterplot matrix graph showing correlations between selected macromechanical properties from DMTA measurements (absolute value of complex modulus, |G*|, and storage modulus, *G*′) and micromechanical properties from MHI experiments (indentation modulus, *E*_IT_, and indentation hardness, *H*_IT_). All values in the plot were measured at an ambient temperature of 22 °C. Diagonal elements of the scatterplot matrix graph show the distribution of the measured quantities, whereas off-diagonal elements show the correlations between the relevant pairs of quantities. The translucent bands around the regression lines represent a 95% confidence interval of the regression estimate. Moreover, all off-diagonal plots show the values of Pearson’s correlation coefficient *r* in the upper left corner and *p*-values in the lower right corner.

Figure 9 compares the stiffness-related properties from the DMTA experiments (the absolute values of the complex modulus, |G*|, and storage modulus *G*′ at 22 °C) and MHI experiments (the values of *E*_IT_ and *H*_IT_ at 22 °C). All correlations are strong (Pearson correlation coefficients *r* close to 1) and statistically significant (*p* values < 0.05). The fact that the properties obtained from the standard *macroscale dynamic experiments* correlate strongly with the properties from the completely independent *microscale quasi-static experiments* confirms the reliability and reproducibility of our measurements and supports the credibility of our interpretations. The extremely strong correlation between |G*| and *G*′ indicates that the prepared TPS composites at 22 °C were mostly solid materials (*G*′ ≫ *G*’’) and, as a result, the contribution of *G*″ to the value of |G*| was small (|G*|^2^ = (*G*′)^2^ + *G*″)^2^). All other correlations between |G*|, *G*′, *E*_IT_, and *H*_IT_ document that all stiffness-related properties of the polymer systems at all length scales were closely interconnected, which agrees with theoretical predictions [46] and re-confirms the reliability and reproducibility of microindentation measurements in polymer systems [49,50]. The theoretical justification for the correlations was given in the studies by Struik [62] (elastic modulus vs. yield stress), Oliver and Phar [46,57] (macro- vs. microscale elastic modulus), and Tabor [58] (yield stress vs. hardness). Similar correlations between macro- and microscale properties have also been observed for amorphous polymers [50], semicrystalline polymers [51], and polymer blends [3,44].

### 3.5. Rheology and Processing Properties

**Figure 10:**
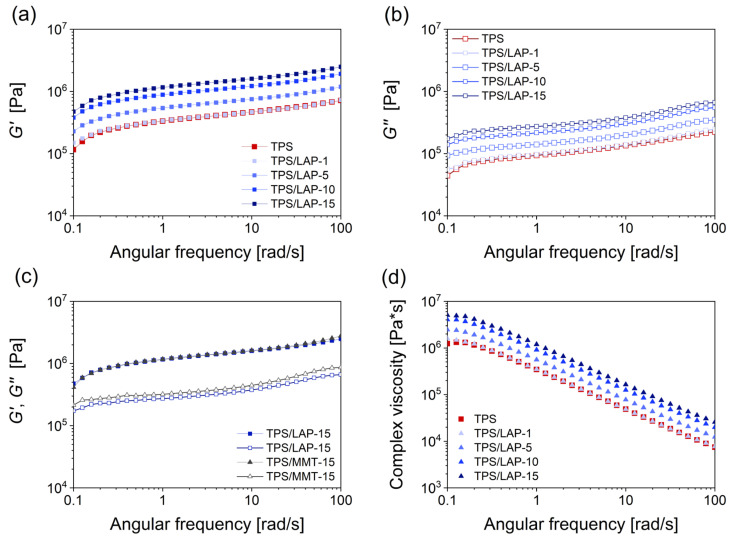
Rheological properties of the studied nanocomposites, with an example being the TPS/LAP samples containing 1, 5, 10, and 15 wt.% LAP measured by oscillatory shear rheometry at 120 °C: the angular-frequency dependence of (**a**) the storage modulus G′, (**b**) the loss modulus G”, (**c**) a comparison by overlaying the G′ and G″ curves for the pair of products containing 15 wt.% LAP or MMT nano-clay, and (**d**) frequency-dependent absolute values of the complex viscosity |η*|, as recorded for the set of samples TPS/LAP.

**Figure 11:**
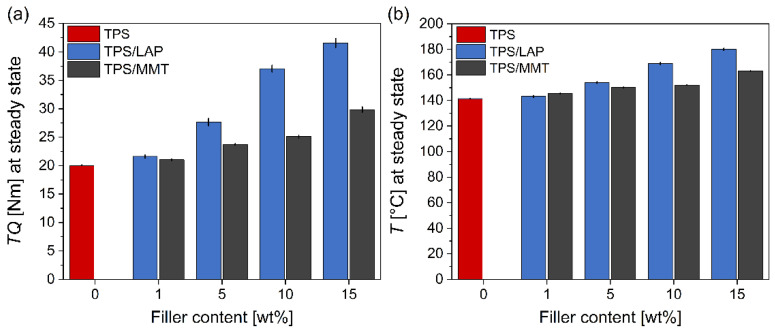
Processing parameters during the melt mixing of TPS/LAP and TPS/MMT systems, which were measured in situ during the sample preparation: (**a**) torque (TQ) and (**b**) processing temperature (*T*_p_). The bar plots show the final average values of TQ and *T*_p_ at a steady state, i.e., after ca. 6 min of melt mixing. The mixing chamber was pre-heated to the nominal processing temperature of 120 °C; the real temperature increased due to the internal friction energy dissipation.

All of the prepared systems were characterized in terms of their basic rheological properties (Figure 10) and of their real processing properties (Figure 11). Their rheological properties were measured by oscillatory shear rheometry using frequency sweep tests at the *nominal processing temperature* of 120 °C (Section 2.9). The real processing properties comprised in situ measurements of *torque* and *real processing temperature* during the melt mixing of the TPS, TPS/LAP, and TPS/MMT systems (Section 2.9).

Figure 10 summarizes the basic rheological characteristics of all of the TPS/LAP nanocomposites and selected TPS/MMT nanocomposites at the nominal processing temperature of 120 °C. The analogous rheological characterization of the TPS/MMT systems is given in Figure S3 in the Supplementary Information. Interestingly, materials with the same filler loadings display nearly identical properties at 120 °C (and at small oscillatory deformations) independently of clay type, MMT, or LAP. Small but still-significant differences were observed between the two nanocomposite families in the previously discussed DMTA profiles (Section 3.4, Figure 8c–d and Figure S2) at 120 °C for filler loadings of 10 wt.%. The near-disappearance of these differences in the frequency sweep tests underlines the dynamic character of the matrix–filler interactions, where continuous oscillatory testing at a constant temperature led to an increased fraction of dissociated physical crosslinks.

In Figure 10a,b, the storage and the loss moduli of all the tested TPS materials display only a moderate frequency-dependent increase: *G*′ increased by 5.8× for neat TPS or 5.1× for TPS/LAP-15; *G*” increased by 5.3× for neat TPS or 3.8× in TPS/LAP-15, while the frequency increased by three orders of magnitude, from 0.1 to 100 rad/s. This might be attributed to a sufficiently fast disconnection and reorganization of the hydrogen bonding in TPS, as well as of the physical crosslinks between TPS and the nano-clay (interaction dynamics) in all studied melts at 120 °C. The minute differences between the curves of the frequency-dependent moduli *G*’ and *G*” for sample pairs with the same filler content (MMT or LAP) are shown in Figure 10c and Figure S4. The absolute values of complex viscosity, |η*|, decreased linearly with the frequency (Figure 10d). This matched the viscosity definition (|G*| = ω|η*|) and the fact that the values *G*′ and *G*″, which define the complex modulus (|G*|^2^ = (*G*′)^2^ + (*G*″)^2^), only exhibited a moderate, quasi-linear increase with the frequency, as discussed above and documented in Figure 10a,b. Within both the TPS/LAP and TPS/MMT series, the viscosity increased systematically with the increasing concentration of the nanofiller. A similar rheological behavior of TPS and its composites has been observed in previous studies, as summarized elsewhere [37,70].

Figure 11 compares the results of the *insitu* measurements of torque (TQ) and processing temperature (*T*_p_) during the melt mixing in a twin-screw laboratory kneader. In Figure 11, the values of TQ and *T*_p_ are shown after ca. 6 min of melt mixing, when the system achieved a steady state (Section 2.9). The addition of both nano-clays raised the torque, which had to be applied in order to maintain a constant rotation rate (Figure 11a), and increased the *real processing temperature* (Figure 11b), which rose from the *nominal processing temperature* of 120 °C to almost 180 °C (the nominal and real processing temperatures are explained in Section 2.9).

Figure 11a summarizes the measured values of torque during the melt mixing. In contrast to the indentation tests (Figure 5), DMTA measurements (Figure 8c,d and Figure S2), and rheological experiments (Figure 10c and Figure S4), the measured TQ values provided evidence that the LAP-filled systems had generated a higher mechanical resistance during the melt mixing, especially at loadings ≥ 5%. This could be explained by a good (in the case of MMT) or even nearly complete (in the case of LAP) exfoliation of the nano-clays. The more-perfectly exfoliated LAP then offered a higher interfacial area for matrix–filler interactions, which in turn led to a higher resistance (internal friction, viscosity) during the melt flow, where these interactions were disrupted (energy consumption via increased torque) and reconnected again (energy release via heat-up) during the process. Importantly, DMTA and rheological tests are based only on small deformations in the linear viscoelastic range, while the sample’s preparation by melt mixing implies continuous material flow. Therefore, the higher effective specific surface area of LAP, which was nearly ideally exfoliated (Figure 2a–c), played a key role during melt mixing that includes intensive continuous flow. On the other hand, the percolating structure of the MMT filler (which is further reinforced by the partial overlapping of the nanoplatelets) (Figure 2d–f) seems to play a dominant role only with small deformations, but it generates much less resistance in the flow state, where the ‘filler network’ is mechanically destroyed, while the smaller specific surface area of MMT stays unchanged. This interpretation is supported by the fact that the strong MMT superstructures were observed to loosen considerably at T > 120 °C (DMTA curves in Figure 8c).

Figure 11b illustrates the increase in the real processing temperature relative to the nominal one (120 °C). With neat TPS, the real processing temperature rises by 20 °C to 140 °C as a consequence of internal friction in the melt. A maximum heat-up effect was achieved with 15% LAP, where the temperature was raised by ca. 60 °C to ca. 180 °C. With both clays, increases in their loading amplified the heat-up effect, but in the case of LAP, the heat-up was more intense, especially at the highest loading. This effect can be attributed to the same causes as the above-discussed torque effect (matrix–filler interface area, degree of exfoliation, dissociation/recombination of matrix–filler interactions). Without the heating-up (melt thinning due to high temperature), the processing torques of the tested nanocomposites would likely be significantly higher.

### 3.6. TGA Investigation of Matrix–Filler Interactions

Thermogravimetric analysis (TGA) yields additional information about the thermal stability and matrix–filler interactions in polymer nanocomposites. In this work, we studied all prepared samples by TGA under both air and nitrogen atmospheres. The TGA experiments documented a stabilizing effect of MMT and LAP, especially at elevated temperatures, where the nano-clays delayed the final oxidation (in air) or carbonization (in nitrogen). The LAP filler was a bit more efficient, which could be attributed to its better exfoliation and dispersion in the polymer matrix. More details concerning the TGA results are given in Section S3 of the Supplementary Information.

### 3.7. Gas Barrier Properties

The nanocomposite TPS/MMT-15, which displayed very attractive mechanical properties and which contained large clay nanoplatelets (sized 100–180 nm vs. LAP: ca. 30 nm), was chosen for the analysis of the gas transport properties. Oxygen, hydrogen, carbon dioxide, and water vapor were used as diffusing gases in these experiments. The results summarized in Table 2 indicate that TPS/MMT-15 displays extraordinarily strong gas barrier properties.

The mentioned dimensions of the studied nanofiller simply mean that the aspect ratio AR of the MMT nanofiller (platelet diameter divided by platelet thickness, which is equal to 1 nm in both cases) is up to six times larger than that of LAP. According to the literature [71,72], mass transport (during gas permeation through a nanocomposite) is indirectly proportional to the aspect ratio of the platelet-type filler. This suggests a six-times-higher efficiency of MMT in the ideal case. In the case of the rheological (mechanical) filler percolation (see DMTA results), the critical filler concentration at which this effect occurs is also inversely proportional to AR (see [73,74]). Both the studied nanofillers, LAP and MMT, were found to percolate between the loadings of 10 and 15 wt.%, which suggests less-than-ideal exfoliation in both cases, and which further confirms that the exfoliation of MMT is less perfect than that of LAP (ideally, MMT should percolate at a markedly lower loading). Nevertheless, the different diameters of the platelets assembled in the percolating filler network remain an important factor: eventual gaps between the ‘crosslinked’ platelets can be reached by a diffusing gas molecule only after it covers a distance approximately equal to the respective platelet width (‘tortuous path’ mechanism, see Nielsen model [72]). This distance is at least six times larger in the case of the percolating (albeit imperfectly exfoliated) nanocomposite filled with 15% of MMT.

The data in Table 2 show that, except for water, extremely low permeabilities were observed for all other gases. The values are of similar order (10^−2^ barrer), while CO_2_ displays the distinctly smallest value (0.035 barrer). Oxygen achieves a ca. two-times-higher, and hydrogen a ca. three-times-higher permeability than CO_2_. In comparison with the literature, e.g., with [75], it can be noted that the permeabilities achieved by TPS/MMT-15 are 20–480 times lower than those in previously published polymer–clay nanocomposites that have been reported to possess good barrier properties. The permeability of TPS/MMT-15 performed similarly well compared to the known gas permeabilities of synthetic butyl rubber for O_2_ and N_2_ [76]. In comparison with [75], the greatest improvement was observed for CO_2_ (480 times), followed by hydrogen (50–100 times) and then oxygen (20 times).

Oxygen diffusion was difficult to measure (it was difficult to distinguish desorption and permeation), and its approximate permeability was found to be similar to that of CO_2_.

Gas permeability is controlled by the diffusion coefficients and solubilities of the respective tested gases in the studied material. Phenomenologically, the size, geometry, and distribution of the impermeable nanofiller, the free volume and eventual interfacial voids in the matrix, and the crystallinity, density, and chain mobility (vs. rigidification), are important factors, as are the chemical affinities. In the case of the studied specimen TPS/MMT-15, apart from the above-discussed large and high-aspect nanofiller, the matrix rigidification (as observed by DMTA) likely also presented an important contribution to its low permeability, as it reduced the formation of temporary voids in the amorphous material (via thermal movements of polymer chain segments). The solubility of the studied gases in TPS played a very important role in the results presented in Table 2: the very high solubility of water in TPS outbalanced its very small diffusion coefficient in TPS/MMT-15 and led to a relatively high permeability. In the case of CO_2_, its affinity with the hydroxyl-rich TPS also markedly raised the solubility of this gas in TPS/MMT-15, but less so than in the case of water, so that the very low diffusion coefficient still led to a spectacularly low permeability. Hydrogen, on the other hand, possesses a very low solubility in TPS, so in spite of its markedly higher diffusion coefficient in comparison to the other gases, its permeability stayed record-low. The result for hydrogen further suggests that interfacial or other permanent voids are not very prominent in the studied material.

## 4. Conclusions

We prepared nanocomposites using thermoplastic starch (TPS) and two common, commercially available nano-clay fillers: Laponite (LAP) and Montmorillonite (MMT). Wheat starch was converted to TPS through our recently developed protocol that comprises solution casting (SC) followed by melt mixing (MM). The LAP or MMT fillers were added to the starch during the first SC step and further dispersed during the second MM step. This preparation protocol yielded biodegradable, highly homogeneous materials with almost perfectly exfoliated nano-clay fillers, with substantially improved mechanical properties and excellent barrier properties. The key features of the prepared materials can be summarized as follows:

1. Both TPS/LAP and TPS/MMT nanocomposites exhibited very homogeneous dispersion and almost perfect exfoliation of unprecedentedly high loadings of the nano-clays, up to 15 wt.%, as evidenced by SEM, TEM, and WAXS measurements.
2. A marked mechanical reinforcement by up to one order of magnitude for MMT and by somewhat less for LAP was observed at room temperature and across several additional temperature ranges, as documented by DMTA measurements.
3. The mechanical reinforcement of the TPS matrix was consistently higher for the TPS/MMT composites than for TPS/LAP (as evidenced by DMTA), but the effect was less homogeneous in the microscale (as proven by microindentation measurements and confirmed by SEM and TEM micrographs).
4. The larger MMT platelet size (ca 100–180 nm vs. LAP platelet size of ca. 30 nm), slightly less perfect exfoliation, platelet overlaps, and stacked multi-layers led to a percolating superstructure of MMT platelets and partially exfoliated agglomerates (as suggested by TEM and confirmed by their excellent barrier properties). Moreover, the percolating superstructure seemed to play a key role in improving the macroscale mechanical properties at the highest MMT loading of 15 wt.% (as evidenced by DMTA, microindentation, and tensile experiments).
5. Dynamic viscosity at small deformations increased for both nanofillers (as proven by rheological measurements), which correlated with higher torques and temperatures during melt mixing (as confirmed by in situ process measurements). The increase in torque and processing temperature was lower for MMT and higher for LAP, which could be attributed to the smaller average size and nearly complete exfoliation of LAP, resulting in a higher specific interfacial area for matrix–filler interactions.
6. Extraordinarily good gas barrier properties at high filler loadings were characteristic of TPS nanocomposites with 15% MMT. This, together with the improved mechanical properties of the TPS matrix, made the material highly attractive for packaging applications, particularly when considering the natural origin, non-toxicity, biodegradability, and abundance of its components. The affinity of the studied nanocomposites to swelling by water could be remedied by assembling the packaging foil from several layers (which is a common production practice), so that the gas-barrier layer would be protected from both sides by water-repelling layers made from suitable materials.

## Figures and Tables

**Table 1:** List of prepared thermoplastic starch composites.

Composites with Laponite		Composites with Montmorillonite	
Sample ID	Filler Content (wt.%)	Sample ID	Filler Content (wt.%)
TPS/LAP-1	1	TPS/MMT-1	1
TPS/LAP-5	5	TPS/MMT-5	5
TPS/LAP-10	10	TPS/MMT-10	10
TPS/LAP-15	15	TPS/MMT-15	15

**Table 2:** Gas transport properties of the specimen TPS/MMT-15 (explanatory notes are below the table).

Gas	Permeability mol·m/m^2^·s·Pa	Permeability Barrer (a)	*D* × 10^11^ (b) m^2^/s	*S* × 10^5^ (c) mol/(m^3^·Pa)
O_2_ (d)	2.098 × 10^−17^	0.062	4.59	0.046
H_2_	2.830 × 10^−17^	0.084	8.491	0.033
CO_2_	1.167 × 10^−17^	0.035	0.912	0.128
H_2_O	7.782 × 10^−16^	2.319	0.965	8.066

(a) Barrer is a common non-SI unit employed in the field of barrier properties. $Barrer\;=10^{-10}\frac{{cm}_{STP}^{3}}{{cm}^{2}}\cdot \frac{cm}{s\cdot cmHg}$; $1\;Barrer=3.35\times 10^{-16}\left(mol\cdot m\right)/(m^{2}\cdot s\cdot Pa)$. (b) *D* = diffusion coefficient; (c) *S* = solubility; (d) The values for oxygen are subject to considerable inaccuracy, as it is almost impossible to distinguish between desorption and permeation (the real diffusion coefficient might be smaller).

## Data Availability

The original contributions presented in this study are included in the article/Supplementary Material. Further inquiries can be directed to the corresponding authors.

## Supplementary Materials

The following supporting information can be downloaded at: https://www.mdpi.com/article/10.3390/ma19020347/s1, Figure S1: Thermomechanical properties of TPS/MMT nanocomposites containing 1, 5, 10, and 15 wt.% of MMT, as well as of neat TPS, measured by DMTA in rectangular torsion mode; Figure S2: Thermomechanical properties: Comparison of LAP- and MMT- based nanocomposites at nano-clay loadings of 1, 5, and 10 wt.%; Figure S3: Rheological properties of TPS/MMT nanocomposites containing 1, 5, 10, and 15 wt.% of MMT, as well as of neat TPS, measured by oscillatory shear rheometry at 120 °C; Figure S4: Rheological properties: Comparison of LAP- and MMT- based nanocomposites at nano-clay loadings of 1, 5, and 10 wt.%; Figure S5: TGA traces and DTG curves of the nanocomposites TPS/LAP and TPS/MMT containing 1, 5, 10 and 15 wt.% of clay, as well as of neat TPS.

## Abbreviations

The following abbreviations are used in this manuscript:

TPS	Thermoplastic starch
LAP	Laponite
MMT	Montmorillonite
SC	Solution casting
MM	Melt mixing
SC+MM	Solution casting followed by melt mixing
SEM	Scanning electron microscopy
TEM	Transmission electron microscopy
WAXS	Wide-angle X-ray scattering
LVER	linear viscoelasticity region
DMTA	Dynamic mechanical thermal analysis
MHI	Microindentation hardness testing
