# Supplementary material for: Thermoplastic Starch Composites with Highly Exfoliated Nano-Clay Fillers and Excellent Barrier Properties
Source: Materials (Basel). 2026 Jan 15;19(2):347. doi: 10.3390/ma19020347 (PMC12842834; doi:10.3390/ma19020347)
Supplement: Supplementary file 1 [file materials-19-00347-s001.zip › materials-4073653-supplementary.pdf]

Supplementary Information

Thermoplastic starch composites with highly exfoliated nanoclay fillers and excellent barrier properties

Veronika Gajdosova[1], Beata Strachota[1], Vaclav Pokorny[1], Libuse Brozova[1], Jan Kozisek[1,2], Ewa Pavlova[1], Zdenek Stary[1], Miroslav Slouf[1]*, Adam Strachota[1,]*

[1]*Institute of Macromolecular Chemistry of the Czech Academy of Sciences, Heyrovsky Sq. 2, 162 06 Prague, Czech Republic*

[2]*Charles University, Faculty of Science, Department of Physical and Macromolecular Chemistry, Hlavova 2030/8, 128 40 Prague 2, Czech Republic*

Contents

S1. Thermomechanical properties (DMTA) 2
  S1.1 TPS/LAP systems: Detailed comments 2
  S1.2 TPS/MMT systems: Detailed comments 4
  S1.3 Comparison of TPS/LAP and TPS/MMT systems 7
S2. Rheology and processing properties 8
S3. Thermogravimetric analysis 10

S1. Thermomechanical properties (DMTA)

S1.1 TPS/LAP systems: Detailed comments

The DMTA characteristics of the TPS/LAP nanocomposites are summarized in Fig. 8a,b in the Main Manuscript File. The DMTA analyses of the TPS/MMT nanocomposite compositions are presented in Figure S1 (further below, in this file). The LAP vs. MMT comparisons for the loadings of 1, 5, and 10 wt.% of both fillers are shown in Figure S2, while the comparison for 15 wt.% is illustrated in Fig. 8c,d.

Three glass transitions are characteristic for the neat TPS as well as for the TPS/LAP nanocomposites, **which follow after three plateau-like thermal regions** (see G' = f(T) graphs in Fig. 8a), namely the stiff glassy state, the soft glassy state and the stiff rubbery state. In case of neat TPS or of the sample with 10 wt.% of LAP, a fourth plateau after the third glass transition also is visible, namely the soft rubbery state which precedes the final melting (that was not investigated in Fig. 8). Except for the stiff glassy state, where the effect of both fillers was minimal, in all the remaining plateaus (including the fourth one), both fillers notably, or even markedly raised the moduli. This implies that they were involved in significantly strong interactions with all the phases of TPS, namely with the plasticizer-rich (diluted) starch, with the amorphous starch-rich (plasticizer-deficient) phase, as well as with the partly semicrystalline starch-rich phase.

In the stiff glassy plateau (at $T < -70$°C, see Fig. 8a), nearly negligible differences are observed between all the TPS/LAP materials (including neat TPS) in their G' = f(T) graphs. The contribution of the matrix-filler interactions in this region is small in comparison to the high strength of van-der-Waals forces (generated at least in 85% by TPS) in the fully glassy material.

In the soft glassy plateau at $T_{g2} > T > T_{g1}$ (region from -30 to 0°C), the G' = f(T) graphs (Fig. 8a) indicate a visible reinforcement of the TPS/LAP systems, by a factor up to ca. 2. At 1% of LAP, no significant reinforcement is observed in the mentioned region. A significant but identical reinforcement is observed at 5 and 10% of LAP, while the highest reinforcing effect seen at 15% of LAP is disproportionately high in comparison to 10% of LAP. In the soft glassy plateau, the starch-rich phases of the matrix still are glassy, while the plasticizer-rich domains (in which starch occurs in a diluted state) already are rubbery and display segmental mobility. The marked increase in reinforcement if going from 10 to 15% of LAP suggests, that well-dispersed and exfoliated LAP can generate relatively efficient crosslinking also in the diluted plasticizer-rich phase.

In the stiff rubbery plateau between T_{g2} and T_{g3} (ca. between +70 and +100°C, see G' = f(T) graphs in Fig. 8a), a stronger reinforcement, by up to somewhat less than one order (factor 8.5) is observed in the TPS/LAP systems. All the LAP loadings generate a notable effect here, but practically no difference is observed between 1 and 5 wt.% of LAP. Like in the previous plateau, and also like in the adjacent transition regions (steps in G') around T_{g1} and T_{g2}, the effect of 15% of LAP is disproportionately higher, than in case of 10% of LAP. Above 100°C, the excessive reinforcing effect of 15% of LAP somewhat decreases. In the case of the nanocomposite with 10% of LAP, its initially high reinforcing effect in the stiff rubbery plateau notably and abruptly decreases between 80 and 88°C: The final glass transition at T_{g3} is the sharpest in this system (10% of LAP), in comparison to the other materials in Fig. 8a. A nearly percolating filler arrangement seems to dissociate here, and above 88°C, the nanocomposites reinforced with 1, 5 and 10% of LAP display a nearly identical value and course of G' = f(T), and thus an identical reinforcement. In contrast to that, the most-filled product containing 15% of LAP stays strongly reinforced (by somewhat less than one order relatively to TPS) until the final temperature of 120°C, and its transition at T_{g3} is gradual, like in the remaining samples (except TPS/10LAP). Here, a percolating filler structure appears to be much more stable than with 10% of LAP.

The effect of LAP loading on the **values of the respective glass transition temperatures T_{g1}–T_{g3}** is illustrated by the **tanδ = f(T) curves** in Fig. 8b: The addition of LAP clay generally alters these temperatures, and at the highest LAP contents, a distinct up-shift of the respective glass transitions is systematically observed.

T_{g1} **(**the **glass transition of the plasticizer-rich domains**, see Fig. 8b**)**, is up-shifted continuously by increasing LAP contents if the filler loading is higher than 1 wt.%. (At 1% of LAP, T_{g1} very slightly decreases, by nearly 1°C). With 5% of LAP, T_{g1} increases to -50°C (from -51.5°C in neat TPS). With 10 and 15% of LAP, T_{g1} increases up to -47.6 and -42°C, respectively. In case of the described shifts (addition of 5–15% of LAP), the T_{g1} peak additionally somewhat broadens gradually, and it decreases in height. The shift and broadening of the T_{g1} peak in all the discussed TPS/LAP nanocomposites probably originates in an overlaid tanδ peak, which is generated by an increasingly prominent immobilized (by interaction with filler) fraction of the glycerol-(plasticizer-)rich phase in the TPS matrix. The decrease of the peak height at T_{g1} can be assigned to an increasing relative elastic character as consequence of physical crosslinking via filler-matrix interactions. In the nanocomposite with 1% of LAP, where no reinforcement is observed below +15°C, the filler seems to play just a disordering role at $T = T_{g1}$, which leads to the slight observed down-shift of T_{g1}.

T_{g2} **(**the **glass transition of the amorphous starch-rich phase**, see Fig. 8b**)** displays even more complex trends than T_{g1}: At first, the T_{g2} value drops from +25.3°C (neat TPS) down to +23°C (1 wt.% of LAP, disordering effect of the finely dispersed nanofiller), while the peak height (with 1 wt.% of LAP) visibly drops, thus indicating a simultaneously increased relative elasticity. This might be related to the observation (G' = f(T) curves in Fig. 8a) that at $T = T_{g2}$, a reinforcing effect appears also with 1% of LAP. With 5 and 10% of LAP, the T_{g2} values are up-shifted relatively to neat TPS (as well as relatively to the sample with 1% of LAP), to 28.4°C and 35.3°C, respectively, while the peak height gradually increases but still stays somewhat smaller than in case of neat TPS. This trend indicates an increasing immobilizing effect of increasingly concentrated LAP in the amorphous starch rich phase, as well as a friction-like phenomenon – probably the dissociation (upon deformation) of weak physical interactions matrix-filler, which generates energy absorption and hence also higher tanδ values. An even higher up-shift of T_{g2} occurs, if going from 10 to 15% of LAP: The T_{g2} peak shifts to a somewhat flat summit extending from 44 to 51°C, while the peak height visibly drops. This observation indicates a relatively strong immobilization, as well as an increased prominence of strong filler-matrix interactions. The latter raise the relative elasticity (thus reducing tanδ).

T_{g3} **(**the **glass transition of starch-rich domains which are partly semi-crystalline**, see Fig. 8b**)** displays somewhat similar trends like T_{g2}, but more complex ones: At first (with 1 and 5% of LAP), the position of T_{g3} does not shift (staying at ca. 69.5°C), albeit the peak shape somewhat alters. The peak height at first notably decreases (with 1% of LAP), but with 5% of LAP it significantly increases again but still stays smaller than in case of neat TPS. At the loadings of 10 and 15% of LAP, the position of T_{g3} markedly up-shifts, to 86.3 and to 105°C, respectively, while the peak height drops below the previously observed values (at T_{g3}), especially so with 15% of LAP, where the peak is 50%-high relatively to neat TPS. At 15% of LAP, the T_{g3} peak additionally becomes extremely flat. Similar effects can be assigned to the trends of T_{g3} like in case of T_{g2}, namely an interplay of physical crosslinking, immobilization and friction-like dissociation of matrix-filler interactions. At the highest filler loadings (10 and 15% of LAP), the crosslinking and immobilization clearly dominate. Moreover, at the highest loading of 15%, the partial semi-crystallinity seems to augment the reinforcing effect of the LAP filler, which also can be observed in the G' = f(T) graphs in Fig. 8a.

S1.2 TPS/MMT systems: Detailed comments

The DMTA characteristics of the TPS/MMT nanocomposites are summarized in Figure S1, while DMTA analyses of TPS/LAP are presented in Fig. 8 in the Main Manuscript File. The LAP vs. MMT comparisons for the loadings of 1, 5, and 10 wt.% of both fillers also are shown in Figure S2, while the comparison for 15 wt.% is illustrated in Fig. 8c,d in the Main Manuscript File.

Three glass transitions are characteristic for the TPS/MMT nanocomposites, similarly like in case of the TPS/LAP systems, as well as of neat TPS. The mentioned transitions **follow after three plateau-like thermal regions** (see *G*' = f(*T*) graphs in Figure S1a), namely the stiff glassy state, the soft glassy state and the stiff rubbery state. In the case of neat TPS, or of the sample with 15 wt.% of MMT, a fourth plateau after the third glass transition also is at least partly visible, namely the soft rubbery state which precedes the final melting (that was not investigated in Figure S1). Except for the stiff glassy state, where the effect of both fillers was minimal, in all the remaining plateaus (including the fourth one), both fillers notably, or even markedly raised the moduli. This implies that they were involved in significantly strong interactions with all the phases of TPS, namely with the plasticizer-rich (diluted) starch, with the amorphous starch-rich (plasticizer-deficient) phase, as well as with the partly semicrystalline starch-rich phase.

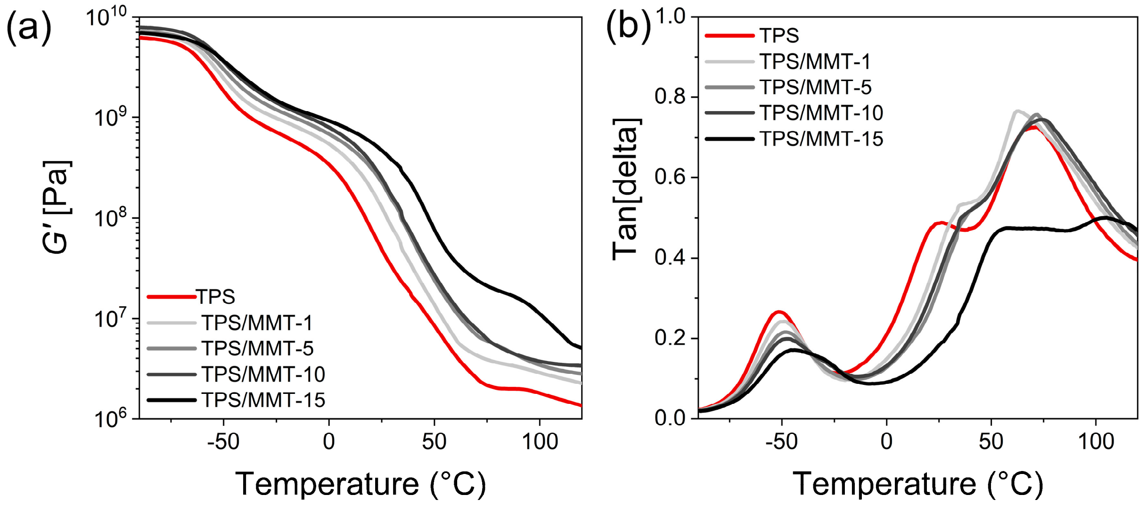

Figure S1. Thermomechanical properties of TPS/MMT nanocomposites containing 1, 5, 10, and 15 wt.% of MMT, as well as of neat TPS, measured by DMTA in rectangular torsion mode, in the temperature range from −90 to +120 °C: (a) storage modulus G′ = f(T); (b) loss factor tan(δ) = f(T).

In the stiff glassy plateau (at *T* < -70°C, see Figure S1a), only small differences are observed between all the TPS/MMT materials (including neat TPS) in their *G*' = f(*T*) graphs, which do not much exceed statistical error. The contribution of the matrix-filler interactions in this region is small in comparison to the high strength of van-der-Waals forces (generated at least in 85% by TPS) in the fully glassy material.

In the soft glassy plateau at $T_{g2} > T > T_{g1}$ (region from -30 to 0°C), the *G*' = f(*T*) graphs (Figure S1a) indicate a reinforcement by up to half an order. In this temperature region, the starch-rich phases of the matrix still are glassy, while the plasticizer-rich domains (in which starch occurs in a diluted state) already are rubbery and display segmental mobility. The reinforcing effect of filler loading in the soft glassy plateau follows a distinctly different trend, if MMT is the filler instead of the above-discussed LAP: With MMT, every increase in filler loading generates a visible additional reinforcement, but in contrast to LAP, the greatest changes occur between neat TPS and the nanocomposites with the lowest MMT loadings, while above 5 wt.% of MMT, the further increase of filler loading (to 10 and to 15%)

leads only to very moderate increments in reinforcement – the reinforcing effect is approaching saturation above 5% of MMT. In contrast to that, 1% of LAP generated a negligible reinforcement in the soft glassy plateau of its composite with TPS, while the most dramatic increase in reinforcement occurred between 10 and 15% of LAP, which in turn is in contrast to MMT, for which between these loadings (10 and 15%) there is only a very small modulus increase. The observed trend seems to be connected with the less than perfect exfoliation of MMT, whose effective dispersion in the plasticizer-rich diluted starch phase seems to reach saturation near 5 wt.% of MMT.

In the stiff rubbery plateau between T_{g2} and T_{g3} (ca. between +70 and +100°C, see G' = f(T) graphs in Figure S1a), a stronger reinforcement, by up to nearly one order can be observed, similarly like in case of LAP. In this plateau, and also in the adjacent transition region (step in G') around T_{g2}, the effect of 15% of MMT is disproportionately higher, than in case of 5 or 10% of MMT. This trend similar to the LAP system and in contrast to the previous G' = f(T) plateau of the MMT system (and to the trend in the region of T_{g1}) where 15% MMT does not display a disproportionate, but just a small incremental additional reinforcement. A contrast to the LAP filler (15% of LAP) is, that above 100°C, the excessive reinforcing effect of 15% of MMT markedly and relatively abruptly drops (sharp T_{g3} transition), albeit it still stays disproportionally high if compared to 10% of MMT. A similar abrupt T_{g3} transition was observed in case of 10% of LAP but not in case of 15% of LAP. A nearly percolating arrangement of the more strongly exfoliated fraction of the MMT filler seems to dissociate above 100°C (this fraction appears to be less prominent than the percolating fraction of LAP in TPS/15%LAP). Hence, TPS/15%MMT behaves similarly like the TPS/10%LAP system. This similarity highlights the less than perfect exfoliation of MMT, which (at T < 100°C) is partly compensated by the larger size of MMT platelets and by their overlapping in the partly exfoliated state. In contrast to the LAP-filled systems, the MMT nanocomposites display different reinforcement even at T > 100°C, albeit the moduli with 1, 5, and 10% of MMT come relatively close to each other. At the highest MMT loading, even after the break-up of the filler percolation, the sample TPS/15%MMT keeps a distinctly higher modulus than TPS/10%MMT. In contrast to MMT, the nanocomposite TPS/15%LAP does not undergo a break-up of the filler percolation even at the highest studied temperature, 120°C.

The effect of MMT loading on the **values of the respective glass transition temperatures T_{g1}–T_{g3}** is illustrated by the **tanδ = f(T) curves** in Figure S1b: The addition of MMT clay up-shifts the temperatures of all these transitions (the trends are simpler than with LAP).

T_{g1} (the **glass transition of the plasticizer-rich domains**, see Figure S1b**)**, is continuously up-shifted by increasing MMT loading, at first (if going from 0 to 10% MMT) gently from -51.5 to -47°C, and with moderate peak broadening, accompanied by a moderate but visible decrease of tanδ values. Finally, if going from 10 to 15 wt.% of MMT, the T_{g1} peak shifts, broadens and decreases in height more dramatically: The flat peak summit of TPS/15%MMT extends between -44 and -31°C, and the peak height decreases by 36% relatively to neat TPS. The shift and broadening of the T_{g1} peak in all the discussed TPS/MMT nanocomposites probably originates in an overlaid tanδ peak, which is generated by an increasingly prominent immobilized fraction of the glycerol-(plasticizer-)rich phase, in which the starch molecules come into interactions with clay nanoplatelets. The decrease of the peak height (at T_{g1}) can be assigned to an increasing elastic character as consequence of physical crosslinking via filler-matrix interactions.

T_{g2} **(**the **glass transition of the amorphous starch-rich phase**, see Figure S1b**)** is up-shifted more dramatically by the increasing MMT loading: at first from +25°C (T_{g2} of neat TPS) to + 37°C (with 1 wt.% of MMT), after which the tanδ curves at 5 and 10% of MMT stay relatively similar in the region of T_{g2} (small further up-shift of T_{g2} to 38-39°C; the whole tanδ curves with 5 and 10% of MMT are very similar). The tanδ value at T_{g2} increases by 10% if going from neat matrix to 1% MMT. Upon raising the

MMT content to 5 or 10% (where nearly identical curves are observed), the tanδ value of the peak summit slightly decreases (by 4%). A dramatic change occurs, however, if going from 10 to 15% of MMT: The T_{g2} peak shifts to a much higher temperature (by at least 26°C), and it transforms into a broad summit plateau extending between +58 and +71°C. The tanδ value at T_{g2} of TPS/15%MMT drops by 9% relatively to the tanδ value observed with 5 or 10% of MMT (at T_{g2}), which is below the tanδ value of the neat matrix at T_{g2}. The amorphous starch-rich phase appears to be at first (with 1–10% of MMT) immobilized only to some degree, which situation does not change at MMT contents between 1 and 10%. The moderate increase in tanδ (with 1–10% of MMT vs. neat TPS) indicates a moderately increased relative plastic character, probably related to weaker matrix-filler interactions, which dissociate upon deformation and thus generate energy absorption. If going from 10 to 15% of MMT, a strong immobilization is generated (additional vast up-shift of T_{g2}) and the prominence of strong filler-matrix interactions obviously increases, thus raising the relative elasticity (which leads to a drop in tanδ).

T_{g3} (the **glass transition of starch-rich domains which are partly semi-crystalline)** displays similar trends like T_{g2}: At first a slight increase (by 6%) in tanδ peak value, if going from neat TPS to the nanocomposite with 1% MMT, after which a small drop in the peak height (by 3%) follows for 5 and 10% MMT, but tanδ at T_{g3} still remains higher than in case of the neat TPS. The temperature of T_{g3} only moderately shifts at these loadings of MMT: from 70°C (neat TPS) down to 63.2°C (1%MMT), then up to 71.5 and 74.7°C (at 5 and 10% of MMT, respectively). After that, a dramatic shift in the T_{g3} position as well as in the tanδ value at T_{g3} is observed, if going from 10 to 15% MMT: T_{g3} is up-shifted by 34°C (from 70 to 104°C), relatively to the neat matrix, while the tanδ value drops by 31% (relatively to neat TPS). Similar effects can be assigned to the trends of T_{g3} like in case of T_{g2}. Additionally, it seems that MMT (as seen at 1%) initially somewhat disturbs the order in the partly semi-crystalline domains of TPS (drop in T_{g3}). At the highest loading of 15%, the partial semi-crystallinity seems to augment the reinforcing effect of the filler, which also can be observed in the G' = f(T) graphs in Figure S1a.

S1.3 Comparison of TPS/LAP and TPS/MMT systems

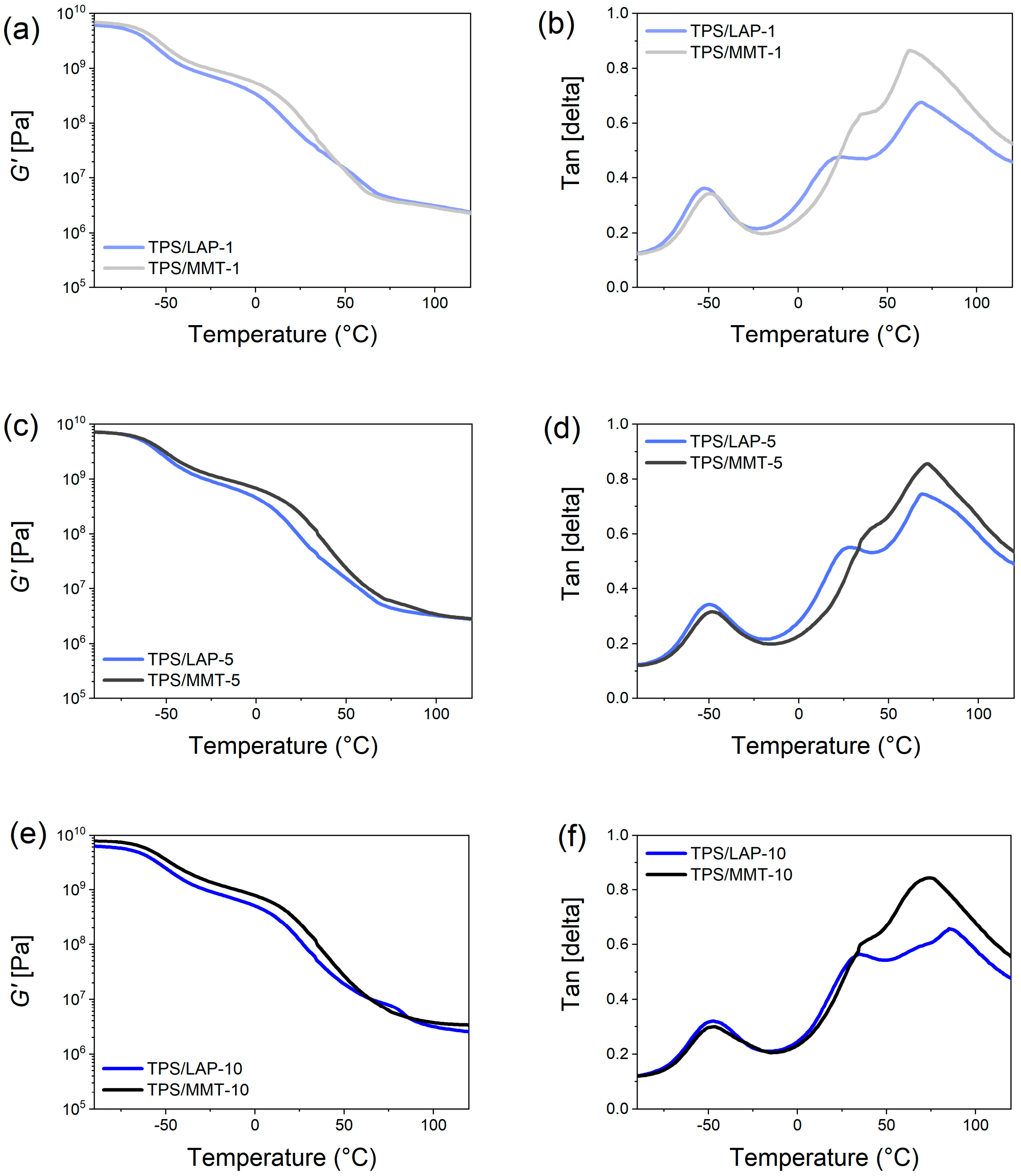

Figure S2. Thermomechanical properties: Comparison of LAP- and MMT- based nanocomposites at nano-clay loadings of 1, 5, and 10 wt.%: In each of the individual graphs, curves of product pairs with the same filler loadings (LAP and MMT) are compared: (a,c,e) G′ = f(*T*); (b,d,f) tan(δ) = f(*T*); DMTA in rectangular torsion mode, in the temperature range from −90 to +120 °C; the comparison at 15% of filler loading is shown in Fig. 8c,d in the Main Manuscript file.

S2. Rheology and processing properties

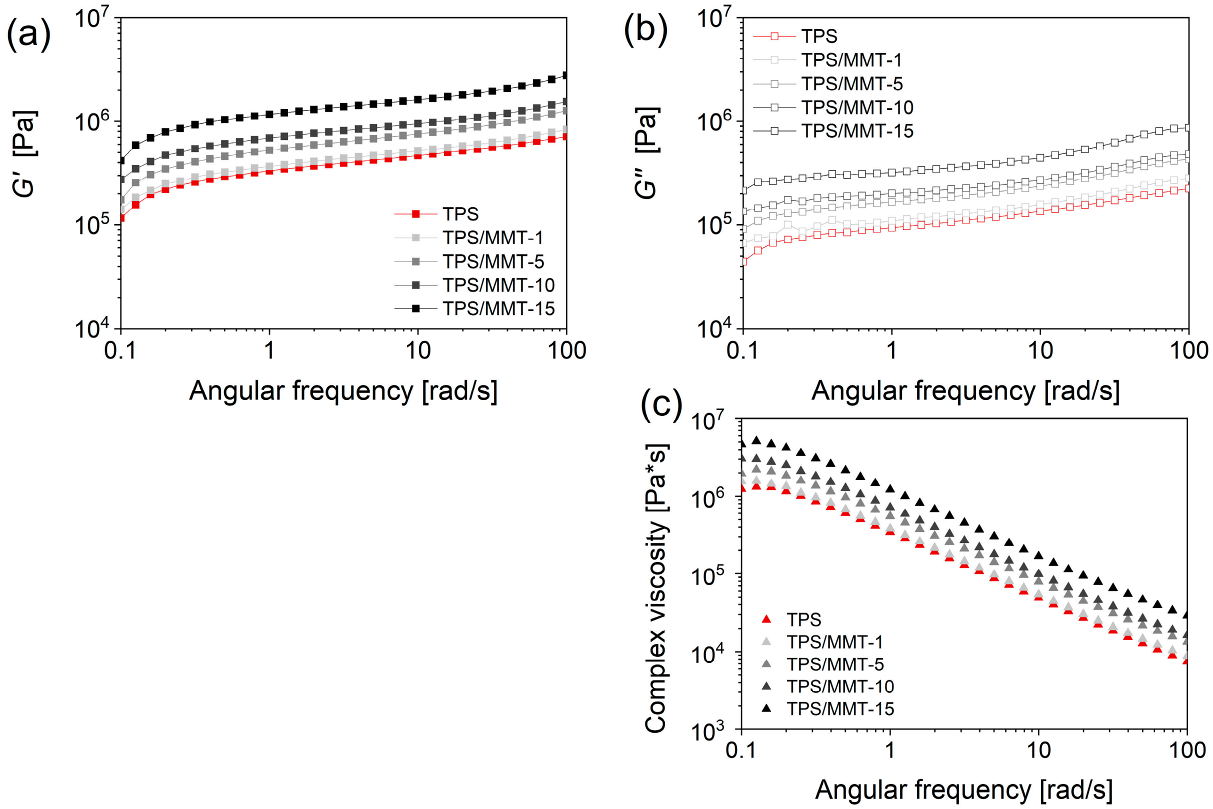

Figure S3. Rheological properties of TPS/MMT nanocomposites containing 1, 5, 10, and 15 wt.% of MMT, as well as of neat TPS, measured by oscillatory shear rheometry at 120 °C: angular-frequency-dependence of: (**a**) storage modulus G', (**b**) loss modulus G'', (**c**) of the absolute values of the complex viscosity $|\eta^*|$.

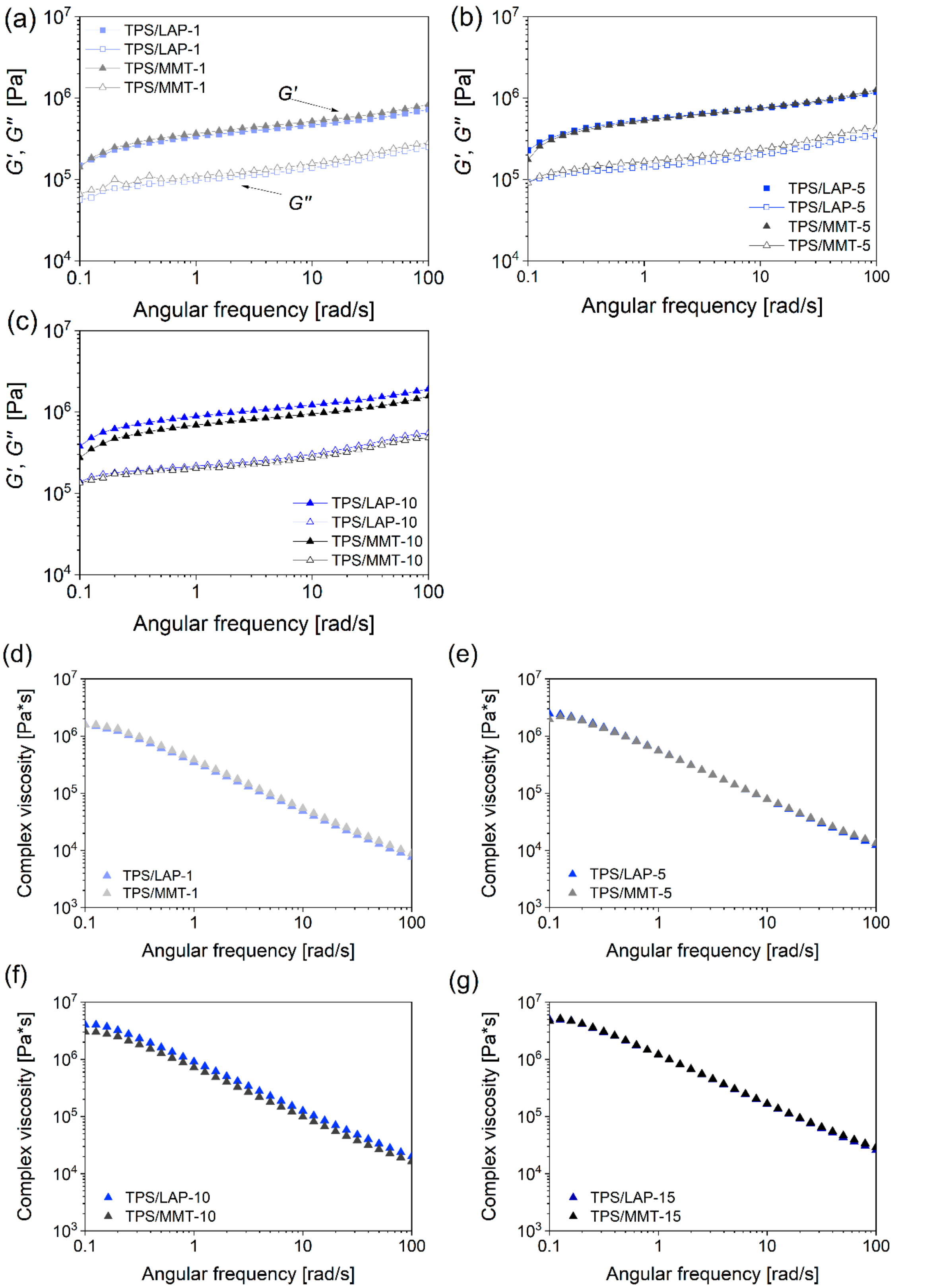

Figure S4. Rheological properties: Comparison of LAP- and MMT- based nanocomposites at nano-clay loadings of 1, 5, and 10 wt.%: In each of the individual graphs, curves of product pairs with the same filler loadings (LAP and MMT) are compared: (a,b,c): G' = f(angular frequency) together with the far-separated G" = f(ang. freq.) for each product pair; (d,e,f,g): absolute values of the complex viscosity $|\eta^*|$ = f(ang. freq.) for each product pair; the comparison of the curves of G' and G" at 15% of filler loading is shown in Fig. 10c in the Main Manuscript file.

S3. Thermogravimetric analysis

Thermogravimetric analysis (TGA) can supply valuable information about matrix–filler interactions in nanocomposites, and about their implications for oxidation- and thermolysis-resistance of these materials. In Fig. S5, the TGA traces (as well as their derivatives) are compared, which were recorded for both the studied families of TPS/clay nanocomposites, in air and in nitrogen, respectively.

Both in air and in nitrogen, all the systems display two small and flat weight loss steps (-15 and -7%, respectively), which in sequence of rising temperature (up to 225 °C, and up to 275 °C, respectively) correspond to the loss of water absorbed in the TPS matrix, and of the water adsorbed on the clay fillers. The main, thermolysis-initiated decomposition step occurs always at ca. 320 °C (loss of ca. 60% of TPS mass), independently of the atmosphere. In air, an extended temporary shoulder is observed in the TGA trace (between 325 and 650–700 °C), which corresponds to char formation by oxidative crosslinking. This shoulder is followed by complete oxidation at higher temperatures (above 500 °C), that leaves only the clay filler as the final ash fraction. In nitrogen, a significant carbonized fraction (9.1% of the original TPS mass) is observed after the main decomposition step (and no additional features). This carbonized fraction, together with eventually present clay, forms the final thermolysis residue in nitrogen.

It can be observed in Fig. S5, that in the final stages of the TGA tests, at $T > 340$ °C, both the clays appear to have a stabilizing effect on the pyrolyzed material, by visibly delaying the final oxidation or carbonization. The stabilizing effect is moderately but visibly higher in case of LAP (see stacking of TGA curves). In case of the tests in nitrogen, this can lead to visibly higher final char fractions: A higher portion of TPS is carbonized in TPS/10%LAP, or in TPS/15%LAP, than in neat TPS itself. The stabilizing effect of the clays is even more markedly illustrated by the dTGA peaks of the main decomposition step: In the most extreme comparison, neat TPS vs. TPS/15%LAP, the mentioned decomposition rate (peak at 322 °C) decreases from -2.30 %/K down to -0.77 %/K in air, or from -2.15 %/K down to -0.81 %/K in nitrogen, respectively. If MMT and LAP are compared, then it can be noted that LAP is more efficient: it reduces the decomposition rate down to 60–40% of the rate achieved with the same loading of MMT clay. The MMT clay still reduces the decomposition rate down to 70% of its value observed with neat TPS in nitrogen (independently of filler loading), or, in air atmosphere, down to 74–57% of the TPS value (at 1–15% of MMT respectively). MMT slightly upshifts (by 1 or 2 °C) the temperature of the decomposition maximum, while LAP down-shifts (by up to 11 °C) and broadens the temperature region of the main decomposition step. The slowing-down of the main decomposition step also is visible, if the slopes of the TGA curves near 320 °C are carefully observed in Fig. S5.

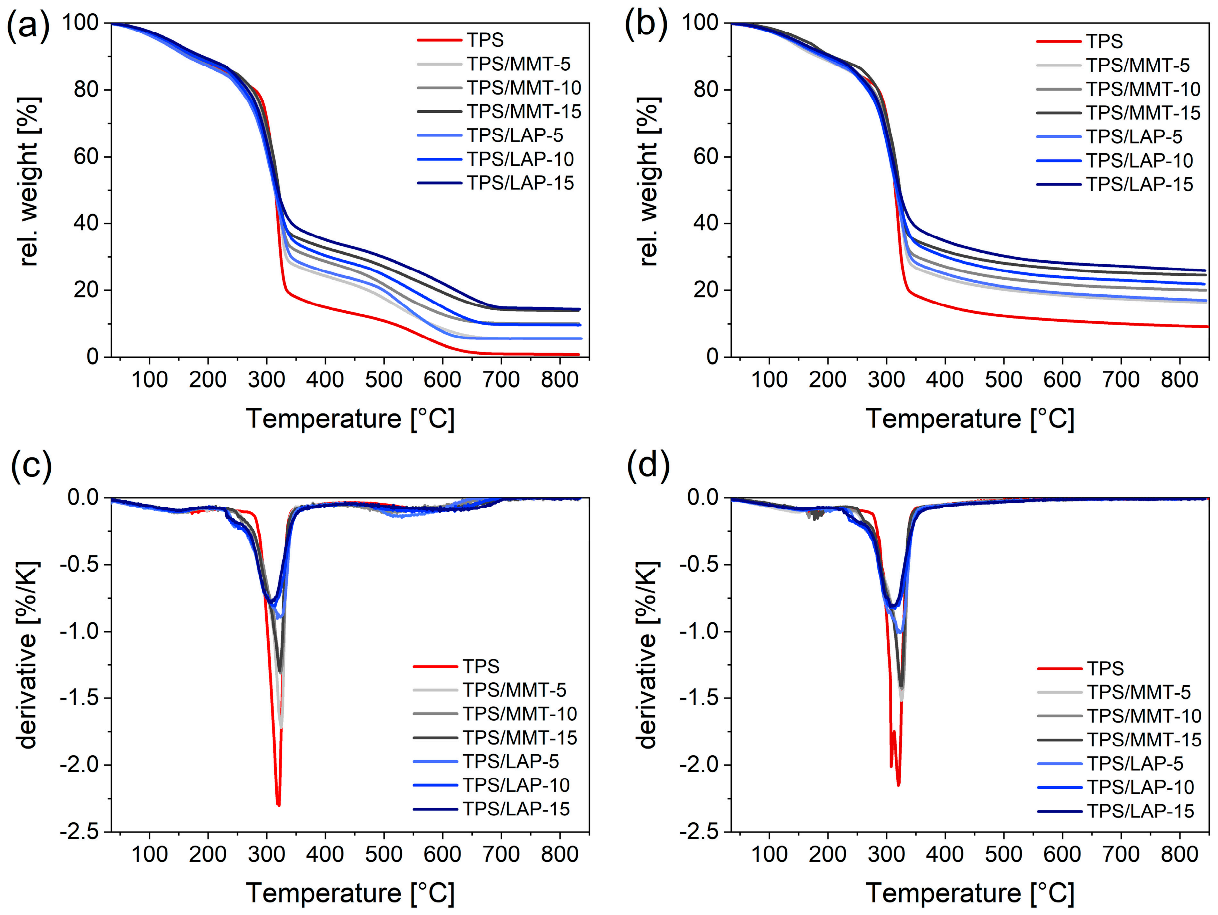

Figure S5. TGA traces (a,b) and DTG curves (c,d) of the nanocomposites TPS/LAP and TPS/MMT containing 1, 5, 10 and 15 wt.% of clay, as well as of neat TPS as a reference: (a,c) analyses in air, and (b,d) analyses in N_2 atmosphere.
